# Expansion of Foxp3^+^ T-cell populations by *Candida albicans* enhances both Th17-cell responses and fungal dissemination after intravenous challenge

**DOI:** 10.1002/eji.201343604

**Published:** 2014-02-13

**Authors:** Natasha Whibley, Donna M MacCallum, Mark A Vickers, Sadia Zafreen, Herman Waldmann, Shohei Hori, Sarah L Gaffen, Neil A R Gow, Robert N Barker, Andrew M Hall

**Affiliations:** 1 Division of Applied Medicine, University of AberdeenAberdeen, UK; 2 Aberdeen Fungal Group, University of AberdeenAberdeen, UK; 3 Academic Transfusion Medicine Unit, Scottish National Blood Transfusion ServiceAberdeen, UK; 4 Department of Pathology, NHS GrampianAberdeen, UK; 5 Sir William Dunn School of Pathology, University of OxfordOxford, UK; 6 Research Unit for Immune Homeostasis, RIKEN Research Center for Allergy and ImmunologyYokohama City, Japan; 7 Division of Rheumatology and Clinical Immunology, University of PittsburghPittsburgh, PA, USA

**Keywords:** *Candida albicans*, Disseminated infection, Foxp3, Regulatory T (Treg) cell, Th17

## Abstract

*Candida albicans* remains the fungus most frequently associated with nosocomial bloodstream infection. In disseminated candidiasis, the role of Foxp3^+^ regulatory T (Treg) cells remains largely unexplored. Our aims were to characterize Foxp3^+^ Treg-cell activation in a murine intravenous challenge model of disseminated *C. albicans* infection, and determine the contribution to disease. Flow cytometric analyses demonstrated that *C. albicans* infection drove in vivo expansion of a splenic CD4^+^Foxp3^+^ population that correlated positively with fungal burden. Depletion from Foxp3^hCD2^ reporter mice in vivo confirmed that Foxp3^+^ cells exacerbated fungal burden and inflammatory renal disease. The CD4^+^Foxp3^+^ population expanded further after in vitro stimulation with *C. albicans* antigens (Ags), and included at least three cell types. These arose from proliferation of the natural Treg-cell subset, together with conversion of Foxp3^−^ cells to the induced Treg-cell form, and to a cell type sharing effector Th17-cell characteristics, expressing ROR-γt, and secreting IL-17A. The expanded Foxp3^+^ T cells inhibited Th1 and Th2 responses, but enhanced Th17-cell responses to *C. albicans* Ags in vitro, and in vivo depletion confirmed their ability to enhance the Th17-cell response. These data lead to a model for disseminated candidiasis whereby expansion of Foxp3^+^ T cells promotes Th17-cell responses that drive pathology.

## Introduction

Fungi are a major cause of nosocomial bloodstream infection, particularly in critically ill patients receiving antibiotics, immunosuppressive treatments or who have undergone surgery 1,2. Approximately 20% of infections found in intensive care unit patients, and 11% of all bloodstream infections, are caused by *Candida* species, of which *Candida albicans* remains the most frequently isolated 1–3. Although *C. albicans* induces innate and adaptive immune responses, mortality rates remain high and a better understanding of factors limiting protective immunity will be critical for the development of more effective therapies 1,3. One such factor may be the balance between host CD4^+^ T effector (Teff) and regulatory T (Treg)-cell responses.

During pathogenic *C. albicans* infection, the immune response is driven by inflammatory mediators, particularly inflammasome-derived IL-1β, and is characterized by the production of IFN-γ from Th1 cells and IL-17A from Th17 cells 4,5. In disseminated *C. albicans* infection, Th1 cells are associated with protection from disease, while a predominance of Th2 cells promotes susceptibility 5. Less clear are the roles of Th17 cells. On the one hand, Th17-cell inflammatory responses appear critical to protective immunity, since mice deficient in IL-17A signaling are particularly susceptible to disseminated *C. albicans* infection 6. On the other, an excessive inflammatory response induced by Th17 cells in mice may cause immune pathology associated with *C. albicans* survival and dissemination 7–9.

It is well established that Treg cells can play a pivotal role in controlling immune responses to microbes 10–12. They are characterized by the expression of Foxp3, a transcription factor that is critical for their development and the most specific marker available for their identification 13. Treg cells also constitutively express high levels of activation markers including CD25 and glucocorticoid-induced TNF receptor-related protein (GITR) 14. Two subsets of Foxp3^+^ Treg cells have now been identified. Natural Treg (nTreg) cells are generated in the thymus, whereas induced Treg (iTreg) cells differentiate from Teff cells in response to antigens (Ags) in the periphery 15,16. Their roles may be further complicated by the recently described phenomenon of plasticity, with Treg and Th17 cells exhibiting interchangeable or overlapping phenotypes 17,18. In addition, Foxp3 can be induced in T cells with effector, rather than regulatory functions 19,20.

Treg cells can potentially have opposing roles during infections, for example, either as a mechanism of immune evasion 21 or by suppressing immune pathology to enhance microbial clearance 22. The overall effect of Treg cells in candidiasis is therefore hard to predict. In murine models of gastrointestinal 23 or oral 11 *C. albicans* infection, increased numbers of Treg cells have been associated with protection from disease, and patients with autoimmune polyendocrine syndromes who have defective Treg cells are susceptible to chronic mucocutaneous candidiasis 24. In contrast, the expansion of a CD4^+^CD25^+^ population, containing Treg cells, inhibited macrophage-mediated innate clearance of *C. albicans* in a murine model of disseminated infection 25. However, whether Treg cells also influence *C. albicans*-induced adaptive Th responses in disseminated infection remains unknown.

The aims of this work were to determine whether the numbers of Foxp3^+^ T cells alter in a murine model of disseminated *C. albicans* infection, and how they contribute to disease. We report that *C. albicans* drives expansion of a complex Foxp3^+^ T-cell population, which is detrimental to the host, since its numbers correlate with fungal burden, and selective depletion in vivo ameliorated pathology. The population exhibited both Treg and Th17-cell functions in vitro, and expanded due to both proliferation of the preexisting nTreg-cell subset and conversion of cells that were previously Foxp3^−^ to Foxp3^+^ iTreg cells, or to intermediate Foxp3^+^/Th17-cell phenotypes.

## Results

### Disseminated *C. albicans* infection induces the expansion of CD4^+^CD25^+^Foxp3^+^ Treg cells

**Figure 1:**
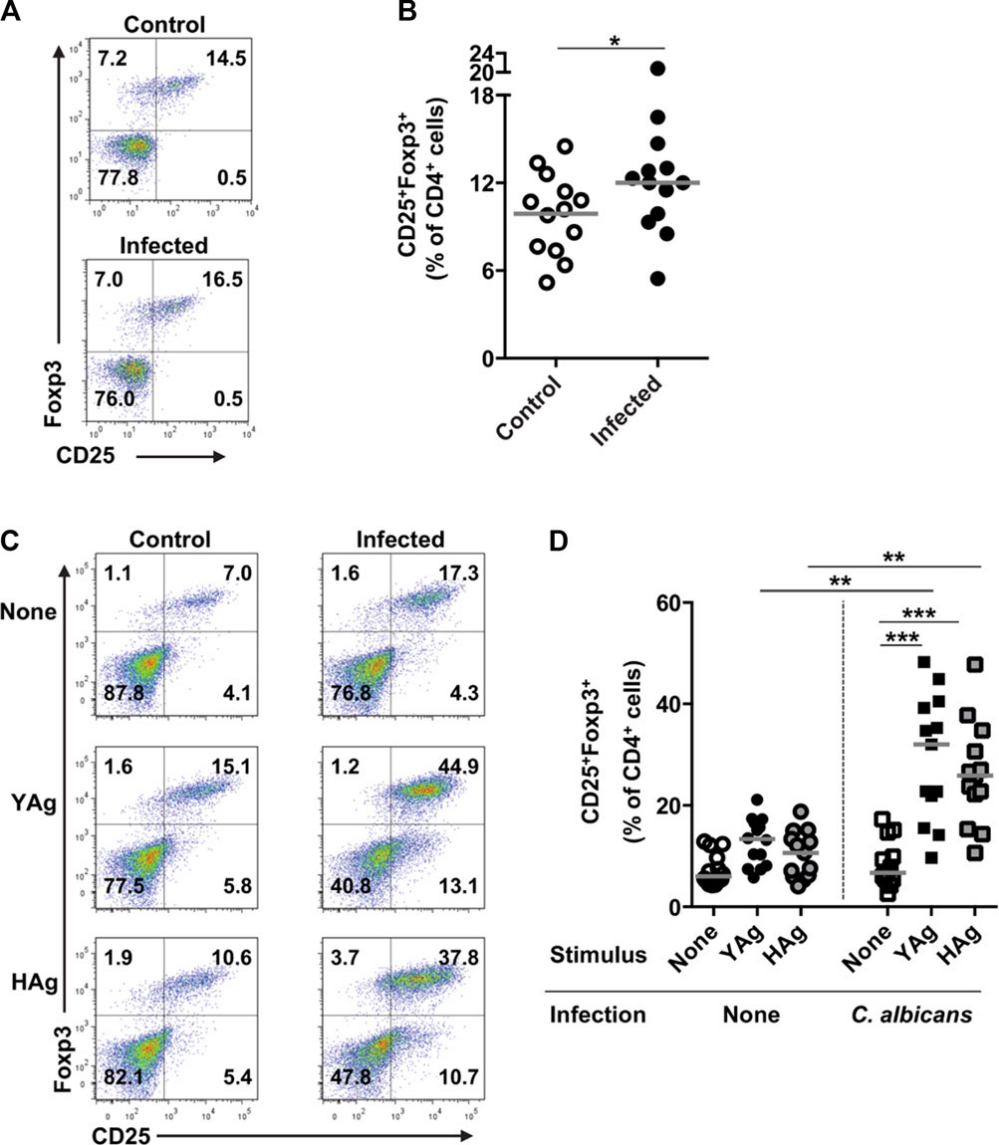
Disseminated *C. albicans* infection is associated with the expansion of cells with a CD4^+^CD25^+^Foxp3^+^ Treg-cell phenotype. (A) Representative flow cytometry plots and (B) graph of SMCs ex vivo, taken from uninfected control or *C. albicans*-infected mice and showing expression of CD25 and Foxp3 on CD4^+^-gated cell. Each symbol in (B) represents an individual animal and the median is indicated by the horizontal line. Results are pooled from 13 independent experiments. *p = 0.02 (Wilcoxon signed-rank test). (C) Representative flow cytometric profiles and (D) graph showing the proportions of CD4^+^-gated cells staining for CD25 and Foxp3 in YAg-or HAg-stimulated SMCs, isolated from uninfected control or *C. albicans*-infected mice. Each symbol in (D) represents an individual animal and the median is indicated by the horizontal line. Results are pooled from 14 independent experiments. ***p < 0.001 (Kruskal–Wallis and post hoc Dunn's multiple comparison tests, **p < 0.025 Mann–Whitney *U*-test with a Bonferroni correction). In all flow cytometry analyses, cells were selected by a live cell and CD4^+^ gate.

C57BL/6 mice were injected intravenously with the *C. albicans* clinical isolate SC5314, and the infection was allowed to progress for 7 days. This model 9,26 mimic's invasive candidiasis in patients, including candidemia and multiorgan infection 27. Renal *C. albicans* burdens correlate with other symptoms of disseminated infection and, since the kidney is typically the last organ to clear the fungus, provide a good indication of disease resolution 9,28. In line with our previous publications 9,28, *C. albicans*-infected mice had high kidney fungal burdens (mean log_10_ CFU/g = 4.5 ± 1.5), kidney immune cell infiltration, and weight loss (Supporting Information Fig. 1). Splenic mononuclear cells (SMCs) were isolated from both *C. albicans*-infected and control uninfected mice, and the populations of cells with the Treg-cell phenotype CD4^+^CD25^+^Foxp3^+^ were enumerated by flow cytometry ex vivo. There was a modest but significant increase (*p* = 0.020, Wilcoxon signed-rank test) in the population of CD4^+^CD25^+^Foxp3^+^ SMCs isolated from infected compared with control mice (Fig. 1A and B), and a similar trend was observed in the kidneys (Supporting Information Fig. 2).

To confirm that *C. albicans* directly induces the expansion of CD25^+^Foxp3^+^ T cells, SMCs were stimulated with SC5314 yeast (YAg) or hyphal (HAg) cell wall Ag preparations. A marked increase in the proportion of CD4^+^ cells that expressed the CD25^+^Foxp3^+^ Treg-cell phenotype was observed in SMCs isolated from *C. albicans*-infected mice and stimulated with either YAg or HAg (*p* < 0.001, Kruskal–Wallis and post hoc Dunn's multiple comparison tests) compared with unstimulated cultures (Fig. 1C and D). By contrast, the proportion of CD25^+^Foxp3^+^ T cells did not increase after stimulation of SMCs from uninfected mice with YAg or HAg.

### Foxp3^+^ T cells are associated with *C. albicans* kidney fungal burden

**Figure 2:**
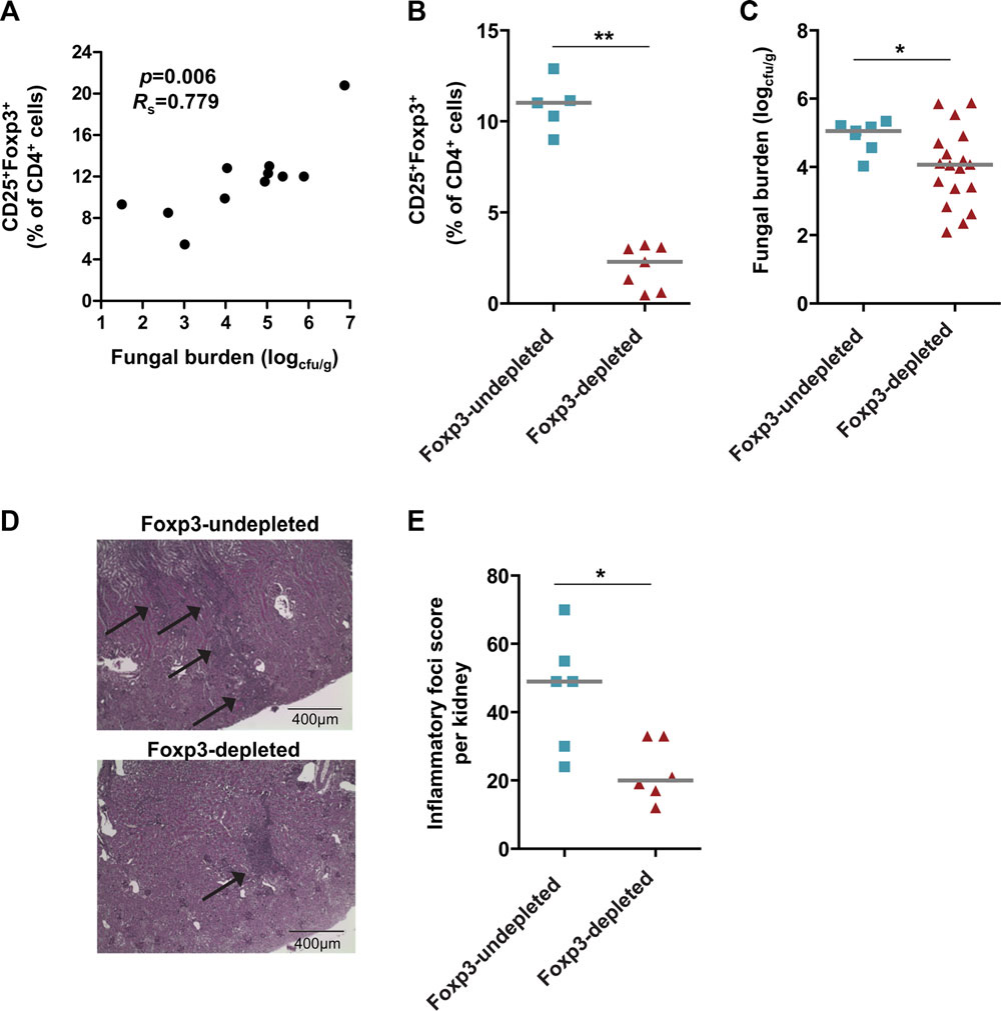
Severity of disseminated *C. albicans* infection is associated with the expansion of Foxp3^+^ Treg cells. (A) Kidney fungal burden and the percentage of CD4^+^-gated cells, isolated from the spleen, expressing both Foxp3 and CD25 in C57BL/6 mice after 7 days of infection with *C. albicans* are shown. Each symbol represents the results from an individual mouse and data are pooled from 11 independent experiments. *p* = 0.006, *R*_s_ = 0.779 (Spearman rank correlation). (B) Proportion of CD4^+^-gated cells expressing CD25 and Foxp3 in peripheral blood of B6.Foxp3hCD2 mice pretreated with anti-hCD2 (Foxp3-depleted), or left untreated (Foxp3-undepleted), 3 days after *C. albicans* infection. Each symbol represents an individual mouse and the horizontal line indicates the median. Data are pooled from five independent experiments. **p < 0.01 (Mann–Whitney *U*-test). (C) Kidney fungal burden 7 days after infection with *C. albicans* in Foxp3-depleted mice compared with Foxp3-undepleted Foxp3^hCD2^ mice. Each symbol represents an individual mouse and the horizontal line indicates the median. Data are pooled from at least seven independent experiments, *p < 0.05 (Mann–Whitney *U*-test). (D) Representative hemotoxylin and periodic acid Schiff stained longitudinal sections of kidneys taken from *C. albicans*-infected, Foxp3-undepleted, or Foxp3-depleted Foxp3^hCD2^ mice 7 days after infection (10× objective, scale bar 400 μm). The arrows mark areas of inflammatory infiltrate in the kidney cortex. Images are representative of six independent experiments. (E) Number of kidney inflammatory lesions with lymphocytic infiltrate in Foxp3-depleted compared with Foxp3-undepleted B6.Foxp3hCD2 mice on day 7 after infection. Each symbol represents an individual mouse and the horizontal line indicates the median. Data are pooled from six independent experiments. *p < 0.05 (Mann–Whitney *U*-test). In all flow cytometry analyses, Foxp3^+^CD25^+^ cells were selected by a live cell and CD4^+^ gate.

The in vivo expansion of Foxp3^+^ T cells in the model of disseminated candidiasis correlates positively (*p* = 0.006, *R*_s_ = 0.779) with kidney fungal burden (Fig. 2A), suggesting that T cells with a Treg-cell phenotype may be associated with the progression of infection. To confirm this, we exploited the ability to deplete most Foxp3^+^ cells from B6.Foxp3^hCD2^ reporter mice that have been genetically modified to co-express human CD2 (hCD2) with Foxp3 19,29. The numbers of Foxp3^+^ T cells can be rapidly and markedly reduced in these mice by a single intraperitoneal injection of anti-hCD2 Ab (Fig. 2B) 29. We deliberately chose the most potent depleting regimen, which efficiently removes the cells for up to 7 days, after which their numbers recover (Supporting Information Fig. 3). When Foxp3^+^ T cells were depleted (Foxp3-depleted) prior to intravenous administration of *C. albicans*, there was a significant decrease in kidney fungal burdens after 7 days compared with B6.Foxp3^hCD2^ mice that had not been pretreated with anti-hCD2 (Foxp3-undepleted; *p* = 0.049, Mann–Whitney *U*-test; Fig. 2C). Depletion of Foxp3^+^ T cells was also associated with fewer kidney inflammatory lesions compared with control Foxp3-undepleted mice (*p* = 0.030, Mann–Whitney *U*-test, Fig. 2D and E).

### *Candida albicans*-infected mice Treg cells suppress Th1-and Th2-type, but enhance Th17-type, responses

**Figure 3:**
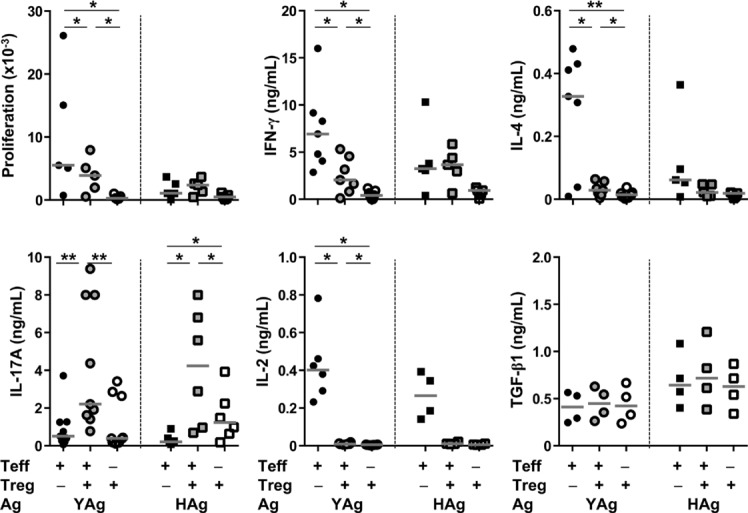
*Candida albicans*-driven Foxp3^+^ Treg-phenotype cells suppress protective Th1-and Th2-type responses, but enhance Th17-cell activation. Teff and Treg cells were isolated from the spleens of C57BL/6 mice, 7 days after infection with *C. albicans*, and cultured with either YAg or HAg in the presence of BM-derived dendritic cells as a source of APC. The graphs show proliferation, measured by ^3^H-thymidine incorporation, and production of IFN-γ, IL-4, IL-17A, IL-2, or TGF-β1, measured by ELISA, Treg-phenotype cells, Treg-depleted Teff cells, or mixed Treg-phenotype and Teff cells (1:1). Each symbol represents an individual mouse and the horizontal line indicates the median. Data are pooled from seven independent experiments, *C. albicans* *p < 0.05, **p < 0.01 (Kruskal–Wallis and post hoc Wilcoxon rank tests).

A defining feature of Treg cells is their ability to inhibit Teff-cell responses, and so it was necessary to determine whether the *C. albicans*-driven population with the Treg-cell phenotype was functionally suppressive in vitro. To test this, the CD4^+^CD25^+^ cell population containing Treg cells (>90% Foxp3^+^), and residual Treg-depleted Teff cells, was fractionated from the spleens of *C. albicans*-infected mice, then cultured with BM-derived dendritic cells, as a source of APCs, and YAg or HAg (Fig. 3). Cultured without Treg cells, Teff cells proliferated and produced IFN-γ, IL-4, IL-17A, and IL-2 in response to YAg stimulation, with IFN-γ predominating. HAg generally stimulated less strong proliferative and cytokine responses than YAg in cultures of Teff cells alone. Addition of the Treg-cell fraction to Teff-cell cultures resulted in significant reductions in proliferation (*p* = 0.031, Kruskal–Wallis and post hoc Wilcoxon rank tests) and production of IFN-γ (*p* = 0.016) and IL-4 (*p* = 0.016) in response to YAg. Strikingly, a significant increase in IL-17A production was observed when Treg-phenotype cells were added to Teff-cell cultures stimulated with either YAg (*p* = 0.004) or HAg (*p* = 0.016). Increased IL-17A production has previously been associated with the consumption of IL-2 by Treg cells 11,30, a cytokine which, together with TGF-β1, is required to stabilize the Treg-cell phenotype 31. The results demonstrate an almost complete loss of IL-2 when Treg-phenotype cells were added to Teff-cell cultures stimulated with either YAg or HAg. However, there was no significant change in TGF-β1 levels in cultures containing purified Teff, Treg, or Teff plus Treg cells. It was also notable that isolated Treg-cell phenotype cells secreted IL-17A when stimulated with either YAg or HAg, albeit at levels that were lower than those seen in co-cultures with Teff cells.

These results revealed that, paradoxically, the Treg-cell phenotype cells suppressed Th1 and Th2 responses in vitro, but, enhanced production of the Teff-cell cytokine IL-17A. Such functional diversity suggested that the expanded population was heterogeneous in origin and/or phenotype, and prompted further characterization.

### *Candida albicans* drives preexisting Foxp3^+^ T-cell proliferation and their induction from effector Th cells

**Figure 4:**
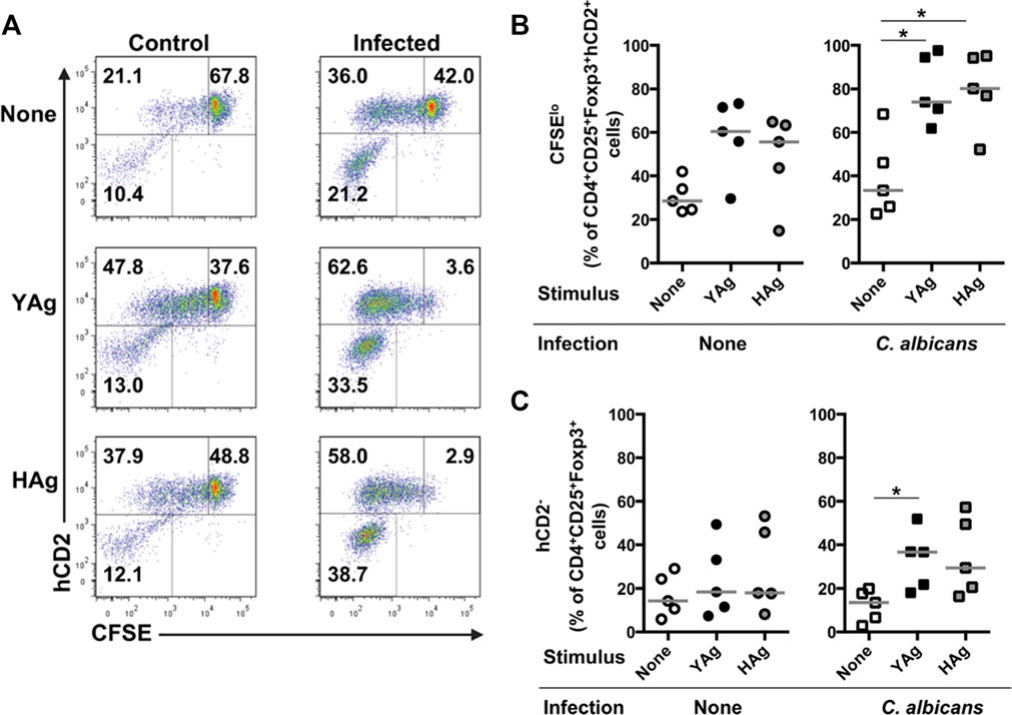
*Candida albicans* drives both the proliferation of preexisting Foxp3^+^ Treg-phenotype cells and their conversion from Teff cells. (A) SMCs were isolated from control uninfected or *C. albicans*-infected mice, 7 days after administration of inoculum or saline, and stimulated with YAg or Hag for 5 days, then stained for hCD2 (preexisting Foxp3^+^ T-cell phenotype) and CFSE (proliferating Foxp3^+^ T-cell phenotype). Representative flow cytometry plots (*n* = 3) showing CD4^+^CD25^+^Foxp3^+^-gated cells are shown. Plots are subdivided into three gates: upper right (CFSE^hi^) = undivided preexisting (hCD2^+^) Treg-cell phenotype, upper left (CFSE^lo^) = dividing preexisting (hCD2^+^) Treg-cell phenotype, lower left = Teff cells that have recently acquired Foxp3 expression. (B) SMCs were isolated from control uninfected or *C. albicans*-infected mice 7 days after administration of inoculum or saline, and stimulated with YAg or HAg for 5 days and the proportion of CD4^+^CD25^+^Foxp3^+^hCD2^+^ preexisting Treg-phenotype cells that are CFSE^lo^ was determined by flow cytometry. Each symbol represents an individual mouse and the horizontal line indicates the median. Data are pooled from five independent experiments, *C. albicans* *p < 0.05 (Kruskal–Wallis and post hoc Dunn's multiple comparison tests). (C) SMCs, collected and stimulated as in (B), were assessed by flow cytometry to determine the proportions of CD4^+^CD25^+^Foxp3^+^ cells that were converted from Foxp3^−^ T cells (hCD2^−^). Each symbol represents an individual mouse and the horizontal line indicates the median. Data are pooled from five independent experiments, *p < 0.05 (Kruskal–Wallis and post hoc Dunn's multiple comparison tests).

Foxp3^+^ T cells can include not only the preexisting thymically derived nTreg-cell subset, but also iTreg cells converted from Teff cells 15,16, and Teff cells that transiently upregulate Foxp3 19,20. The next question was whether the expansion of the CD4^+^CD25^+^Foxp3^+^ T-cell population was due to proliferation of a preexisting Treg-cell phenotype, or conversion from Foxp3^−^ Teff cells. To address this, CD4^+^CD25^+^ Treg cells were isolated from either *C. albicans*-infected or uninfected control B6.Foxp3^hCD2^ mice (preexisting Treg-cell phenotype), labeled with carboxyfluorescein succinimidyl ester (CFSE) and co-cultured with unlabeled, Treg-cell-depleted SMCs from infection-matched WT C57BL/6 mice (Teff cells). Cultures were stimulated with YAg or HAg and analyzed by flow cytometry for the expression of CD4, CD25, Foxp3, hCD2, and CFSE. In these cultures, any Foxp3^+^ cells expressing hCD2 were derived from a preexisting population with a Treg-cell phenotype, with dilution of their CFSE staining indicating proliferation, while those Foxp3^+^ cells that did not express hCD2 were converted from Teff cells. Representative analyses, gated for CD4^+^CD25^+^Foxp3^+^, are illustrated in Figure 4A, and the results are summarized in Figure 4B and C. Following stimulation with either YAg or HAg, there was a substantial increase in the proliferation (CFSE^lo^) of preexisting (hCD2^+^) Foxp3^+^ T cells from infected mice (Fig. 4B). Over 80% of hCD2^+^ cells proliferated in stimulated cultures from infected mice (Fig. 4B), representing approximately 50% of the total Foxp3^+^ population. In addition to driving proliferation of the preexisting Foxp3^+^ fraction, *C. albicans* Ags also significantly increased (*p* < 0.026, Kruskal–Wallis and post hoc Dunn's multiple comparison tests) conversion of cells to a Foxp3^+^ phenotype from hCD2^−^ Teff cells, typically accounting for approximately a third of the total Foxp3^+^ T-cell population in cultures from infected mice (Fig. 4C). These results are consistent with respective contributions by both the nTreg-cell subset, and by cells with either iTreg-or intermediate Foxp3^+^ Teff-cell phenotypes.

### Expression of additional Treg-cell markers by Foxp3^+^ T cells expanded by *C. albicans*

**Figure 5:**
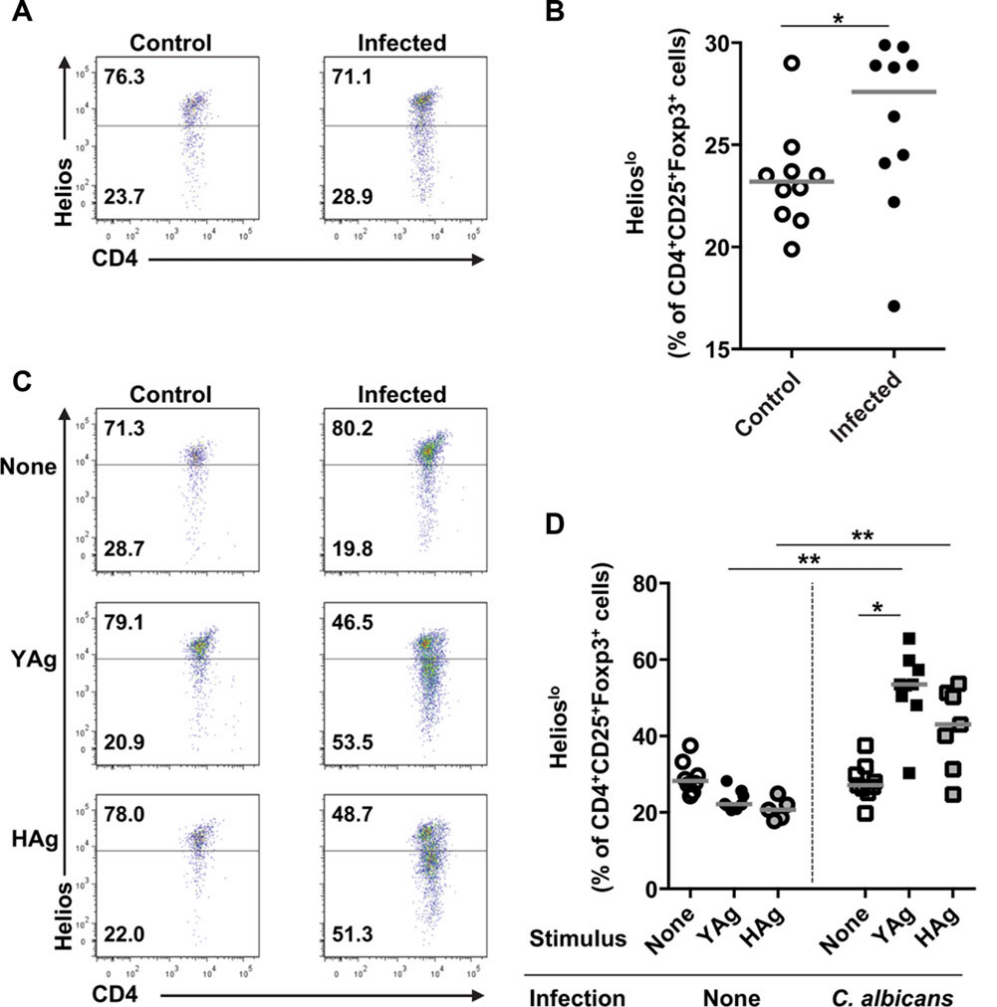
*Candida albicans* drives the expansion of Foxp3^+^Helios^lo^ T cells. (A) Representative flow cytometry plots and (B) proportions of CD4^+^CD25^+^Foxp3^+^-gated SMCs, isolated from control uninfected or *C. albicans*-infected mice 7 days after administration of inoculum or saline, that are Helios^hi^ or Helios^lo^. In (B), each symbol represents an individual mouse and the horizontal line indicates the median. Data are pooled from ten independent experiments, *p < 0.05 (Mann–Whitney *U*-test). (C) Representative flow cytometry plots and (D) proportions of CD4^+^CD25^+^Foxp3^+^-gated SMCs, isolated from control uninfected or *C. albicans*-infected mice, 7 days after administration of inoculum or saline, that express Helios following 5 days of stimulation with YAg or HAg in vitro. Each symbol represents an individual mouse and the horizontal line indicates the median. Data are pooled from eight independent experiments, *p < 0.05 (Kruskal–Wallis and post hoc Dunn's multiple comparison tests), **p < 0.025 (Mann–Whitney *U*-test with a Bonferroni correction).

**Figure 6:**
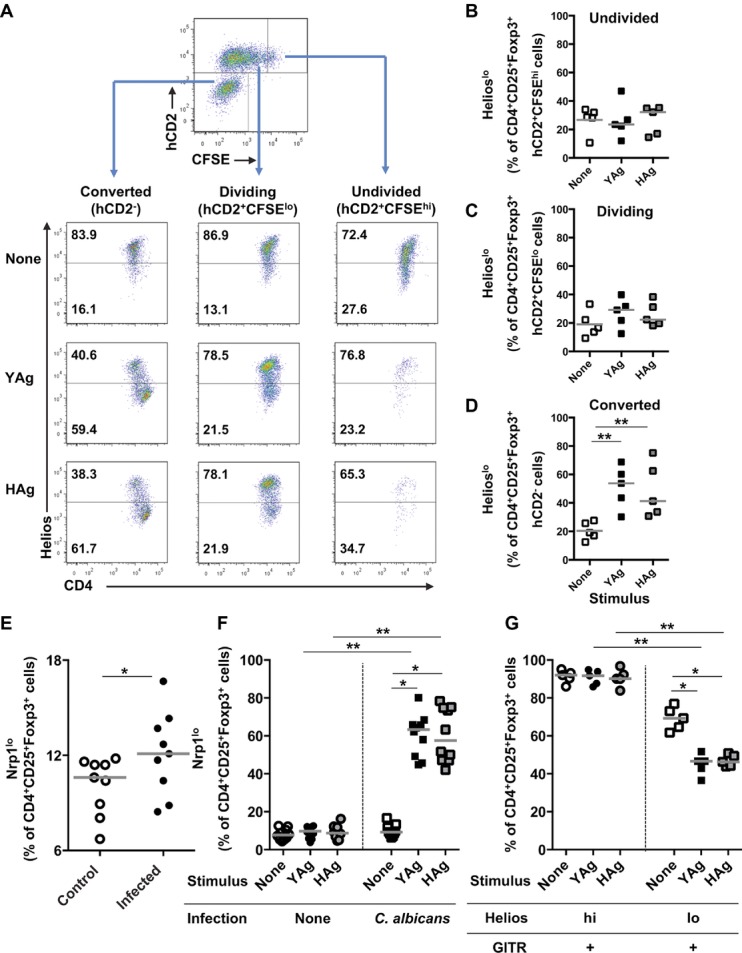
*Candida albicans* induces an increase in the proportion of Foxp3^+^Helios^lo^ cells that have converted from Foxp3^−^ Teff cells. (A) SMCs isolated from *C. albicans*-infected Foxp3^hCD2^ mice, 7 days after administration of inoculum or saline, were stimulated with YAg or HAg for 5 days and then analyzed by flow cytometry (*n* = 5) for the expression of CD4 and Helios in populations of cells gated on dividing precommitted Treg-phenotype (CD4^+^CD25^+^Foxp3^+^hCD2^+^CFSE^lo^), undivided precommitted Treg-phenotype (CD4^+^CD25^+^Foxp3^+^hCD2^+^CFSE^hi^), or Treg-phenotype cells that have converted from Teff cells (CD4^+^CD25^+^Foxp3^+^hCD2^−^). Flow cytometric analyses were performed by sequentially selecting live, CD4^+^, CD25^+^Foxp3^+^, and hCD2^lo/hi^CFSE^lo/hi^ cells, and the gating strategy is depicted in the top panel. (B–D) Summary graphs of populations depicted in (A), showing the proportion of CD4^+^CD25^+^Foxp3^+^ Treg-phenotype SMCs, that are Helios^lo^ after gating on (B) undivided precommitted Treg (CD4^+^CD25^+^Foxp3^+^hCD2^+^CFSE^hi^), (C) dividing precommitted Treg (CD4^+^CD25^+^Foxp3^+^hCD2^+^CFSE^lo^), or (D) cells that have converted from Teff cells (CD4^+^CD25^+^Foxp3^+^hCD2^−^). In (B–D), each symbol represents an individual mouse and the horizontal line indicates the median. Data are pooled from five independent experiments. **p < 0.01 (Kruskal–Wallis and post hoc Dunn's multiple comparison tests). (E) The proportion of CD4^+^CD25^+^Foxp3^+^ cells that express low levels of Nrp-1, ex vivo, from *C. albicans*-infected or control C57BL/6 mice, 7 days after administration of inoculum or saline, was determined by flow cytometry. Live, CD4^+^, CD25^+^Foxp3^+^, and Helios^lo^ cells were selected for the expression of Nrp-1. Each symbol represents an individual mouse and the horizontal line indicates the median. Data are pooled from eight independent experiments, *p < 0.05 (Mann–Whitney *U*-test). (F) SMCs were isolated from *C. albicans*-infected or control mice, 7 days after administration of inoculum or saline, and stimulated with YAg or HAg for 5 days. The proportion of CD4^+^CD25^+^Foxp3^+^ cells expressing low levels of Nrp-1 was assessed by flow cytometry. Each symbol represents an individual mouse and the horizontal line indicates the median. Data are pooled from ten independent experiments, *p < 0.05 (Kruskal–Wallis and post hoc Dunn's multiple comparison tests), **p < 0.025 (Mann–Whitney *U*-test with a Bonferroni correction). (G) Cells were isolated from *C. albicans*-infected mice 7 days after administration of the inoculum and stimulated with YAg or HAg for 5 days in vitro. Expression of GITR on CD4^+^CD25^+^Foxp3^+^Helios^hi^ and CD4^+^CD25^+^Foxp3^+^Helios^lo^ cells was assessed by flow cytometry. Flow cytometry analyses were performed by sequentially selecting live, CD4^+^, CD25^+^Foxp3^+^, and Helios^lo/hi^ cells for the expression of GITR. Each symbol represents an individual mouse and the horizontal line indicates the median. Data are pooled from five independent experiments, *p < 0.05 (Kruskal–Wallis and post hoc Dunn's multiple comparison tests), **p < 0.025 (Mann–Whitney *U*-test with a Bonferroni correction).

The transcription factor Helios is reportedly highly expressed by the nTreg-cell, but not the iTreg-cell, subset 32, nor by Foxp3^+^ Teff cells 19. The levels of Helios in Foxp3^+^ SMCs were determined (Fig. 5). Although the majority of CD4^+^CD25^+^Foxp3^+^ cells were strongly positive for Helios, there was significant expansion of CD4^+^CD25^+^Foxp3^+^Helios^lo^ cells from infected mice (*p* = 0.027, Wilcoxon rank test) ex vivo (Fig. 5A and B), and after stimulation with *C. albicans* Ags (*p* < 0.05 Kruskal–Wallis and post hoc Dunn's multiple comparison tests) in vitro (Fig. 5C and D). When cells from the co-culture experiments depicted in Figure 4 were analyzed for Helios expression, a large majority (>70%) of the dividing (CFSE^lo^) and nondividing (CFSE^hi^) preexisting (hCD2^+^) Foxp3^+^ Treg cells expressed the Helios^hi^ nTreg-cell phenotype. Following stimulation with YAg or HAg, there were significant increases in the Helios^lo^ proportions of the newly converted (hCD2^−^) Foxp3^+^ T cells, but not of the dividing or nondividing preexisting Foxp3^+^ T cells that are hCD2^+^ (Kruskal–Wallis and post hoc Dunn's multiple comparison tests; Fig. 6A–D).

Since the reliability of Helios to identify nTreg cells has been questioned 33, we also stained cells for neuropilin 1 (Nrp-1), which has recently been shown to be a more consistent marker of this subset 34,35. Analysis of Nrp-1 staining on CD4^+^CD25^+^Foxp3^+^ cells expanded by *C. albicans* ex vivo (Fig. 6E) or in vitro (Fig. 6F) demonstrated similar trends of expression to those for Helios. We also examined the expanded populations for the additional Treg-cell marker GITR 14, since Teff cells may be induced to express Foxp3 without acquiring the full Treg-cell phenotype and function 19,20. As expected of nTreg cells, essentially all the CD4^+^CD25^+^Foxp3^+^Helios^hi^ cells stained for GITR (Fig. 6G). However, fewer of the CD4^+^CD25^+^Foxp3^+^Helios^lo^ cells expressed this marker of a full Treg-cell phenotype, with approximately 70% GITR^+^ when cultured from mice infected with *C. albicans*, typically falling to less than 50% after stimulation of SMCs with YAg or HAg (Fig. 6G).

Taken together, our interpretation of the results is that *C. albicans* expands at least three populations of Foxp3^+^ T cells, including proliferation of committed nTreg cells and the de novo expression of Foxp3 by distinct T cells with either iTreg-or Teff-cell phenotypes.

### A minor proportion of the expanded Foxp3^+^Helios^lo^ T cells express Th17-cell markers

**Figure 7:**
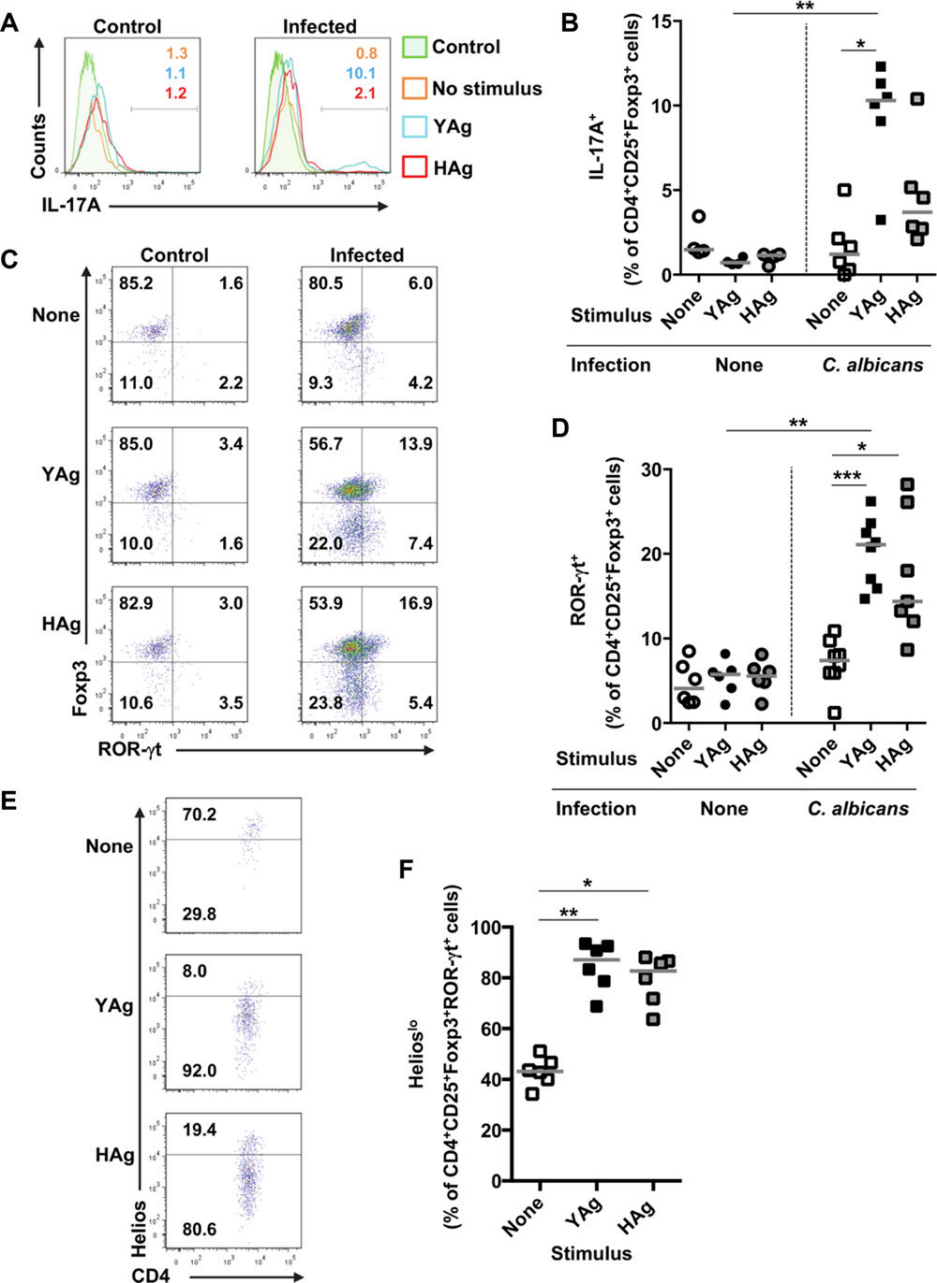
The majority of *C. albicans*-driven Foxp3^+^ T cells that co-express ROR-γt are Helios^lo^. (A) Representative flow cytometry plots and (B) expression of intracellular IL-17A in CD4^+^CD25^+^Foxp3^+^-gated SMCs, isolated from control uninfected or *C. albicans*-infected mice, 7 days after administration of inoculum or saline, and stimulated with YAg or HAg for 5 days in vitro. Each symbol represents an individual mouse and the horizontal line indicates the median. Data are pooled from six independent experiments, *p < 0.05 (Kruskal–Wallis and post hoc Dunn's multiple comparison tests), **p < 0.025 (Mann–Whitney *U*-test with a Bonferroni correction). (C) Representative flow cytometry plots and (D) graph showing the expression of Foxp3 and ROR-γt in CD4^+^CD25^+^-gated SMCs, isolated from control or *C. albicans*-infected mice 7 days after administration of inoculum or saline, and stimulated with YAg or HAg for 5 days in vitro. Each symbol represents an individual mouse and the horizontal line indicates the median. Data are pooled from eight independent experiments, *p < 0.05, ***p < 0.001 (Kruskal–Wallis and Dunn's post hoc multiple comparison tests), **p < 0.025 (Mann–Whitney *U*-test with a Bonferroni correction). (E) Representative flow cytometry plots and (F) proportions of CD4^+^CD25^+^Foxp3^+^ROR-γt^+^ SMCs that are Helios^lo^, isolated from *C. albicans*-infected mice 7 days after administration of inoculum, and stimulated with YAg or HAg for 5 days in vitro. Each symbol represents an individual mouse and the horizontal line indicates the median. Data are pooled from six independent experiments, *p < 0.05, **p < 0.01 (Kruskal–Wallis and post hoc Dunn's multiple comparison tests).

One explanation for the association observed between Foxp3^+^ T cells and Th17 cells in *C. albicans*-infected mice would be the presence of intermediate phenotypes 17, or transient expression of Foxp3 in Teff cells 19,20. Flow cytometric analyses (Fig. 7A and B) demonstrated that the CD4^+^CD25^+^Foxp3^+^ populations responding to YAg included minor proportions of cells (up to 12%) expressing IL-17A, and that the numbers of these Foxp3^+^IL-17A^+^ cells were significantly higher than in unstimulated cultures (*p* < 0.05, Kruskal–Wallis and post hoc Dunn's multiple comparison tests). Furthermore, a similar proportion of the CD4^+^CD25^+^ cell populations that expanded in response to YAg-or Hag-contained cells co-expressing the respective Treg-and Th17-cell transcription factors Foxp3 and ROR-γt (Fig. 7C and D). Most (∼80%) of these cells with the CD4^+^CD25^+^Foxp3^+^ROR-γt^+^ intermediate phenotype expressed low levels of Helios following stimulation with YAg or HAg, consistent with recent induction of Foxp3 expression (Fig. 7E and F). The vast majority (>95%) of the CD4^+^CD25^+^Foxp3^+^ROR-γt^+^Helios^lo^ cells failed to express the additional Treg-cell marker GITR, indicative of Teff-rather than Treg-cell properties (data not shown).

These analyses confirm that, within the Foxp3^+^ T-cell population expanded by *C. albicans*, the fraction converted to become Foxp3^+^ was more heterogeneous than the proliferating, committed nTreg-cell fraction, as it contained a mixture of cells with either iTreg-or Th17-cell characteristics.

### Depletion of Foxp3^+^ cells decreases Th17-cell responses in *C. albicans*-infected mice

**Figure 8:**
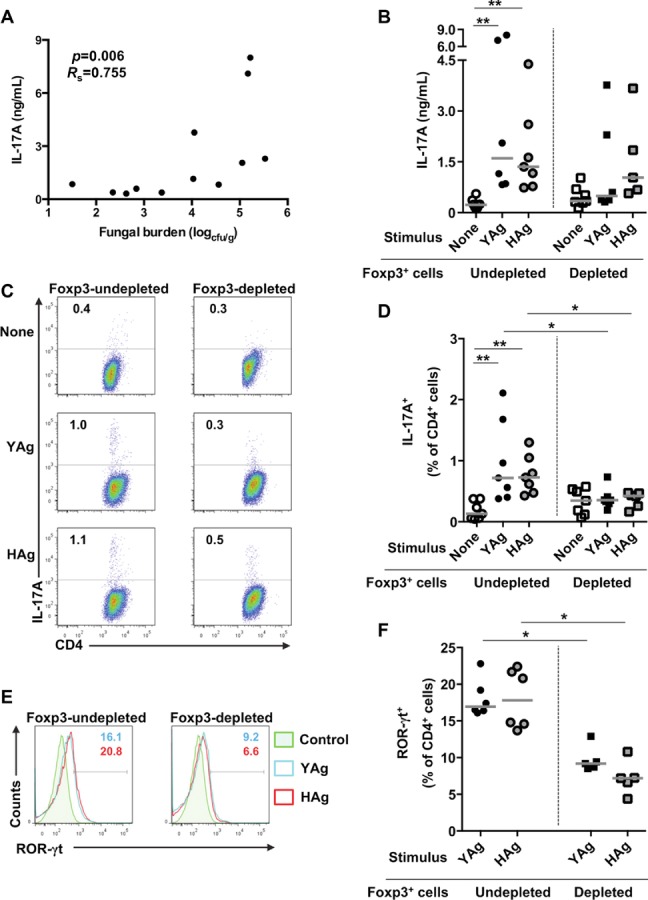
Depletion of Foxp3^+^ T cells reduces Th17-cell responses in *C. albicans-*infected mice. (A) The correlation between kidney fungal burden and IL-17A production by SMCs stimulated with YAg for 5 days in vitro, from *C. albicans*-infected mice 7 days after administration of inoculum, is shown (*p* = 0.006, *R*_s_ = 0.755, Spearman rank correlation). (B) SMCs were isolated from *C. albicans*-infected B6.Foxp3^hCD2^ Foxp3-depleted and Foxp3-undepleted mice, 7 days after administration of inoculum, and stimulated with YAg or HAg for 5 days in vitro. Production of IL-17A was assessed by cELISA. Each symbol represents an individual mouse and the horizontal line indicates the median. Data are pooled from seven independent experiments, **p < 0.01 (Kruskal–Wallis and post hoc Dunn's multiple comparison tests). (C, D) B6.Foxp3hCD2 mice were depleted of Foxp3^+^ T cells and then infected with *C. albicans* for 7 days. SMCs were then stimulated with YAg or HAg for 5 days in vitro and CD4^+^ cells assessed for intracellular IL-17A expression by flow cytometry. Cells were selected by a live cell gate and representative plots are shown in (C). (D) Each symbol represents an individual mouse and the horizontal line indicates the median. Data are pooled from seven independent experiments, **p < 0.01 (Kruskal–Wallis and post hoc Dunn's multiple comparison tests), *p < 0.025 (Mann–Whitney *U*-test with a Bonferroni correction). (E, F) ROR-γt expression was assessed by flow cytometry using YAg-or HAg-stimulated CD4^+^ cells taken from *C. albicans*-infected, Foxp3-undepleted mice. Cells for analysis were selected by a live cell gate and representative flow cytometry plots are shown in (E). (F) ROR-γt expression is shown in both YAg-and HAg-stimulated CD4^+^ cells isolated from Foxp3-depleted versus Foxp3-undepleted mice. Each symbol represents an individual mouse and the horizontal line indicates the median. Data are pooled from six independent experiments, *p < 0.025 (Mann–Whitney *U*-test with a Bonferroni correction).

Excessive Th17-cell responses have been associated with disease progression and immune pathology in a model of gastrointestinal candidiasis 7. Here, there is a positive correlation between kidney fungal burden in disseminated *C. albicans* infection and the ability of SMCs to produce IL-17A in response to fungal Ag in vitro (Fig. 8A). Therefore, one explanation for the exacerbation of *C. albicans* lesions by Foxp3^+^ T cells would be the ability of this expanded population to produce the inflammatory cytokine IL-17A and promote Th17-cell responses, which was demonstrated in vitro. To confirm that Foxp3^+^ T cells also enhance IL-17A responsiveness in vivo, Foxp3^+^ T cells were depleted from B6.Foxp3^hCD2^ mice, or left intact, prior to infection with *C. albicans*. SMCs from infected, Foxp3-undepleted control mice produced significant IL-17A versus unstimulated cells when cultured with either YAg (*p* ≤ 0.01, Kruskal–Wallis and post hoc Dunn's multiple comparison tests) or HAg (*p* ≤ 0.01; Fig. 8B). In comparison, IL-17A production by SMCs from Foxp3-depleted mice was attenuated, with no significant IL-17A produced in response to *C. albicans* Ags. Flow cytometric analyses of both YAg (*p* = 0.018 Mann–Whitney *U*-test with Bonferroni correction) and HAg (*p* = 0.014) stimulated cultures confirmed that there were significantly fewer IL-17A^+^ Th cells when SMCs were taken from Foxp3-depleted versus Foxp3-undepleted *C. albicans*-infected mice (Fig. 8C and D). In parallel with the changes in IL-17A production, staining for the Th17 master transcription factor ROR-γt demonstrated significantly fewer ROR-γt^+^ Th cells in cultures of SMCs isolated from Foxp3-depleted versus Foxp3-undepleted infected mice after stimulation with YAg (*p* < 0.025 Mann–Whitney *U*-test with Bonferroni correction) or HAg (*p* < 0.025; Fig. 8E and F).

One interpretation of these data associating Foxp3^+^ populations, IL-17A responses, and fungal burden is that, while IL-17A responses can provide important protection against *C. albicans* 6, high levels of the cytokine in the context of systemic infection can drive immune pathology and be counterproductive for the host. In support of this hypothesis, IL-17RA KO mice 6, or animals receiving high doses of neutralizing anti-IL-17A Ab, exhibited higher kidney burdens in disseminated infection, with a trend for low levels of the Ab to have a moderate protective effect (Supporting Information Fig. 4A). It is also plausible that the effects of manipulating Foxp3^+^ populations in vivo are mediated through their suppression of IFN-γ responses, reflecting the results shown in vitro (Fig. 3). However, there was no effect of Foxp3^+^ cell depletion in vivo on IFN-γ responses to YAg or HAg (Supporting Information Fig. 4B and C).

## Discussion

This work demonstrates, for the first time, that disseminated infection with *C. albicans* drives the expansion of complex Foxp3^+^ cell populations, including cells with nTreg-, iTreg-, and Th17-cell characteristics. Depletion of Foxp3^+^ cells in reporter mice revealed that the net effect of these populations in vivo is to increase *C. albicans*-associated pathology and fungal burden. These results suggest a model whereby the expansion of Foxp3^+^ T cells promotes Th17-cell responses that drive pathology during disseminated *C. albicans* infection and have implications in the design of immunotherapy to target T cells in candidiasis.

Evidence of the potency with which *C. albicans* drives the expansion of cells with a Treg-cell phenotype is provided by the increased numbers of CD4^+^CD25^+^Foxp3^+^ cells in the spleens of infected mice, and the further growth of these populations when stimulated with *C. albicans* Ags in vitro. An increase in this population was also found in the kidney where T-cell populations have been associated with disease progression 36. Distinct, thymically committed nTreg cell, and peripherally generated iTreg-cell subsets are now recognized 15,16 and cells with characteristics of nTreg cells account for much of the expanding subpopulation of Foxp3^+^ T cells. High levels of Helios 32, and more recently Nrp-1 34,35, have been reported to distinguish the nTreg-from the iTreg-cell subset, although there is some controversy 33 over the reliability of these markers that may reflect Foxp3 stability rather than identify distinct lineages 37. Cells expressing these markers predominated in the enlarged Foxp3^+^ splenic T-cell population from animals infected with *C. albicans*, and proliferated further in SMCs cultures challenged with YAg or HAg.

In addition to nTreg cells, cells with iTreg-cell characteristics made a contribution to the expansion directed by *C. albicans*. These cells were derived from Foxp3^−^ Teff cells that converted to Foxp3^+^ cells in response to *C. albicans* Ags, and expressed the Helios^lo^ 32 and Nrp-1^lo^ 34,35 phenotypes attributed to iTreg cells. Virtually all the Helios^hi^, and many of the Helios^lo^, Foxp3^+^ T cells expressed GITR as a marker of the full Treg-cell phenotype, and the regulatory function of the Foxp3^+^ populations containing these cells was confirmed by their ability to suppress Th1-and Th2-type responses in vitro. Although not previously reported in disseminated fungal disease, dual expansion of nTreg and iTreg cells follows bacterial colonization of the gut 10, and the two cell types may play different roles in infection. For example, nTreg cells may suppress protective immunity while iTreg cells control pathology driven by excessive inflammatory responses 38,39.

Despite its role as the Treg-cell master transcription factor, there is accumulating evidence that induction of Foxp3 expression in Teff cells does not necessarily lead them to adopt the full Treg-cell phenotype or regulatory functions 19,20. Here, the population converted to express Foxp3 by *C. albicans* appeared heterogeneous, or uncommitted, with a minority of cells that, unlike iTreg cells, lacked GITR expression and instead shared characteristics with the Th17-cell subset. Foxp3^+^ T cells, isolated from infected mice, not only produced IL-17A in vitro when stimulated with *C. albicans* Ags, and enhanced Th17-cell effector responses, but they also included a fraction that co-expressed Foxp3 together with the signature Th17-cell transcription factor ROR-γt. Although Foxp3 can directly suppress ROR-γt and IL-17A expression 40, murine and human studies have shown that Foxp3^+^ Treg cells can indeed co-express ROR-γt and/or IL-17A 17,41. Furthermore, activation of Teff cells can lead to transient upregulation of Foxp3 without the adoption of regulatory properties 19,42. In human studies, IL-17A production is enriched within the iTreg-cell population 32,41, although the iTreg cells that produce IL-17A can retain a suppressive phenotype 18. One explanation for these complex phenomena is that Treg cells require stable Foxp3 expression to adopt a “true” suppressive phenotype 20,31. Both TGF-β1 15 and IL-2 31 are required to drive the conversion from a Teff cell to a fully committed, suppressive iTreg cell, and stable Foxp3 expression may be compromised by the low levels of IL-2 that we observed when T cells responded to *C. albicans*. Consumption of IL-2, or inhibition of its production, by Treg-cell populations has also been reported to enhance Th17-cell generation 30, a phenomenon that may contribute to the ability of Treg cells to increase IL-17A production during Teff-cell responses to *C. albicans*.

During infectious disease, Treg cells can potentially inhibit microbial clearance, or benefit the host by suppressing immune pathology 21,22. We took advantage of a Foxp3 reporter mouse 19,29 to determine the overall balance between such properties in mice infected systemically with *C. albicans*. The finding that depletion of Foxp3^+^ cells reduced both kidney fungal burden and the number of inflammatory lesions indicates that, in the numbers induced by *C. albicans*, their net effect is detrimental to the host. This confirmed the initial observation that Foxp3^+^ cell expansion correlated positively with fungal burden. There are two explanations for such an effect, which are not mutually exclusive. First, the ability of Treg-cell populations from *C. albicans*-infected mice to suppress IFN-γ responses in vitro supports the notion that they contribute to fungal persistence as a mechanism of immune evasion, since Th1 cells can protect against infection 5. However, this explanation is at odds with our in vivo studies, in which depletion of Foxp3^+^ cells did not alter IFN-γ production. Second, the current results demonstrate that Foxp3^+^ T cells support, or exhibit, a Th17-cell phenotype during responses to *C. albicans* and can therefore exacerbate, rather than inhibit, inflammation. This latter effect may not only contribute to immune pathology, but also favor the progress of infection, since excessive inflammatory responses are associated with dissemination of *C. albicans* 7,9.

Our work focuses on the adverse consequences of disseminated candidiasis, but the balance between Treg-and Teff-cell responses and their effects may differ at mucosal sites 12. *Candida albicans* colonization of mucosae can induce the expansion of Treg cells 11, but in such locations they appear to suppress excessive inflammatory responses to enhance control of the pathogen 23. The role of IL-17A in our model calls for comment. Previous studies of disseminated *C. albicans* infection, utilizing IL-17A^−/−^ and IL-17RA^−/−^ mice, have reported that Th17-cell responses are beneficial to the host 6. However, such KO models do not address the possibility that harmful and protective effects of IL-17A may depend on the levels produced, and the location or type of cells responsible. Our data suggest a model whereby pathology results from excessive Th17-cell responses associated with Foxp3 induction in disseminated candidiasis, while not excluding a role for lower levels of IL-17A in protection from the fungus. The consequences of Th17-cell expansion may also depend on the site, with protective effects more clearly outweighing the risks of bystander inflammatory damage in mucosal versus disseminated *C. albicans* infection. Indeed, in contrast to our studies of systemic *C. albicans* challenge, adoptive transfer of CD25^+^ T cells enhanced IL-17-cell production and fungal clearance in a mucosal infection model 11. The susceptibility of Treg-and Th17-cell deficient patients with autoimmune polyendocrine syndromes to chronic mucocutaneous candidiasis, but not disseminated candidiasis 24, also supports the view that different CD4^+^ T-cell responses protect against *C. albicans* infection at different locations.

The complexity of the Treg-and Th17-cell responses to *C. albicans* infection raises questions as to the optimal approach for immunotherapy. Treatments that perturb the balance between different CD4^+^ T-cell subsets may inadvertently provide an environment that allows the dissemination of the fungus and exacerbates disease. However, cells with intermediate iTreg/Th17-cell properties may represent new therapeutic targets.

## Materials and methods

### Mice and infections

C57BL/6 (Harlan) and B6.Foxp3^hCD2^ mice 19,29 were maintained at the University of Aberdeen. The work was approved by the UK Home Office. Female mice, 6–12 weeks old, were injected with 1 × 10^5^ yeast cells (*C. albicans* clinical isolate SC5314) or sterile saline via the tail vein, weighed daily, and sacrificed 7 days after infection 28. Foxp3^+^ cells were depleted from B6.Foxp3^hCD2^ mice, 1 day prior to administration of *C. albicans*, with an intraperitoneal injection of 1 mg neutralizing anti-hCD2 monoclonal Ab (YTH655) in sterile saline 29.

### Kidney fungal burdens and inflammation

Kidneys were collected, homogenized, and the growth of *C. albicans* colonies quantified on Sabouraud agar plates. Renal inflammatory lesions were scored in hematoxylin and periodic acid Schiff stained sections 28.

### Fungal Ag preparations

Yeast or hyphal cell wall Ag preparations were obtained by disaggregation of SC5314 *C. albicans* cultures, with intracellular proteins removed by fractionation. *Candida albicans* yeast cultures were grown for 18–24 h in NGY medium and incubated at 30°C with continuous rotation. Hyphae were generated by further incubation for 6 h at 37°C in deionized water (purified by Milli-Q) containing 20% FCS. Yeast or hyphal cells were washed in sterile distilled and deionized water and stored at −80°C. Cells were washed twice in 10 mM Tris-HCl (pH 7.5), before glass beads (Sigma) were added. The tubes were shaken vigorously for 30 s to lyse the cells and allowed to cool. This was repeated six times before the remaining supernatant was removed from the beads and washed five times in sterile 1 M NaCl to remove intracellular proteins. The cell wall fraction was then washed three times in sterile water, re-suspended in DMSO and stored at −80°C. Cell wall concentrations were derived from the initial wet cell wall weight. Each Ag was added to cell cultures at a final concentration of 200 μg/mL (wet weight), with diluent alone as negative control.

### Murine cell culture

Spleens were homogenized and then passed through a 40 μm sterile filter (BD, UK). To isolate kidney resident lymphocytes, kidneys were flushed with PBS, homogenized, and then passed through a 40-μm sterile filter. Lymphocytes were isolated over a density gradient (Sigma), washed, and re-suspended in complete media. Cells were cultured at 1.25 × 10^6^ SMCs/mL in complete media consisting of MEM-α medium (Invitrogen) supplemented with 5% FCS, 4 mM l-glutamine, 100 μg/mL penicillin/streptomycin, 1 × nonessential amino acids, 1 mM sodium pyruvate, 50 mM 2-ME with appropriate stimulus for 5 days in a humidified atmosphere of 5% CO_2_. Where required, Treg-phenotype cells and residual Teff cells were fractionated from SMCs with the CD4^+^CD25^+^ Treg-cell isolation kit (Miltenyi Biotech), and each fraction set at 0.5 × 10^6^ cells/mL, either alone or together, for co-culture with BM-derived dendritic cells as a source of APC at 1.25 × 10^6^ cells/mL. To generate BM-derived dendritic cells, the femurs and tibia from healthy mice were flushed, BM cells washed, and then incubated for 10 days in complete medium supplemented with 2.5% Ag8653 GM-CSF-containing supernatant (a gift from Brigitta Stockinger, NIMR, UK). Loosely adherent and nonadherent DC were recovered and enumerated for co-culture with T cells.

### Flow cytometry

CFSE labeling of cells was performed according to manufacturer's instructions (Cayman Chemicals). After fixation, permeabilization and blockade of nonspecific binding (buffers from eBioscience) cells were stained with appropriate combinations of the following antibodies: CD4-FITC (eBioscience), CD4-PerCP (BD), CD25-Pacific Blue (Biolegend) Foxp3-PE (eBioscience), Helios-allophycocyanin (Biolegend), Nrp-1-allophycocyanin (R&D Systems), ROR-γt-PE (eBioscience), and IL-17A-AlexaFluor647 (eBioscience). Cells were incubated with 25 ng/mL PMA, 250 ng/mL ionomycin, and 1 μL/mL brefeldin A (BD) for 5 h prior to intracellular cytokine staining. Samples were acquired on the BD LSR II and analyzed with FlowJo (Tree Star) or FCS express (De Novo) software.

### Proliferation and cytokine measurement

Cell proliferation was estimated by ^3^H-thymidine incorporation in triplicate wells 5 days after stimulation with *C. albicans* Ags. Cell cultures were transferred to triplicate wells on a round bottom 96-well plate and incubated in the presence of 1 μCi 3H-thymidine for 6 h in a humidified atmosphere of 5% CO_2_. Results are presented as the mean cpm of the triplicate wells.

Cytokines were measured by cellular ELISA using monoclonal Ab pairs specific for IFN-γ, IL-4, IL-2, IL-17A, or TGF-β1 (all Pharmingen). Maxisorp plate (Nunc) wells were coated with anticytokine Ab and then blocked with 3% BSA in PBS (pH 7.2). Cell culture sample or recombinant standard were applied to each well and allowed to incubate for 6 h in a humidified atmosphere of 5% CO_2_. The plates were then developed with the appropriate biotinylated monoclonal detection Ab (Pharmingen), ExtraAvidin-alkaline phosphatase conjugate (Sigma), and p-nitrophenyl phosphate substrate (Sigma). The absorbance at 405 nm was measured using a multiscan plate reader (Labsystems, Basingstoke, UK). Cytokine secretion was calculated by interpolation from a standard curve generated by incubating triplicate wells with doubling dilutions of recombinant cytokine standard.

### Statistical analyses

The Mann–Whitney *U*-test, Wilcoxon matched pairs signed-ranks test, Kruskal–Wallis with Dunn's multiple comparison test, Bonferroni correction, or Spearman rank correlation were used to determine significance where appropriate (SigmaPlot, SyStat Software, Graphpad Prism Software).

## Abbreviations

GITR: glucocorticoid-induced TNF receptor-related protein
HAg: hyphal Ag
iTreg: induced Treg
Nrp-1: neuropilin-1
nTreg: natural Treg
SMC: splenic mononuclear cell
Teff: T effector
YAg: yeast Ag
