# Supplementary material for: Expansion of Foxp3+ T-cell populations by Candida albicans enhances both Th17-cell responses and fungal dissemination after intravenous challenge
Source: Eur J Immunol. 2014 Feb 13;44(4):1069–83. doi: 10.1002/eji.201343604 (PMC3992851; doi:10.1002/eji.201343604)
Supplement: Supplementary file 1 [file eji0044-1069-sd1.pdf]

European Journal of Immunology

Supporting Information

for

DOI 10.1002/eji.201343604

Natasha Whibley, Donna M. MacCallum, Mark A. Vickers, Sadia Zafreen, Herman Waldmann, Shohei Hori, Sarah L. Gaffen, Neil A. R. Gow, Robert N. Barker and Andrew M. Hall

Expansion of $Foxp3^+$ T-cell populations by *Candida albicans* enhances both Th17-cell responses and fungal dissemination after intravenous challenge

© 2014 The Authors. *European Journal of Immunology* published by Wiley-VCH Verlag GmbH & Co. KGaA Weinheim.

Supporting information

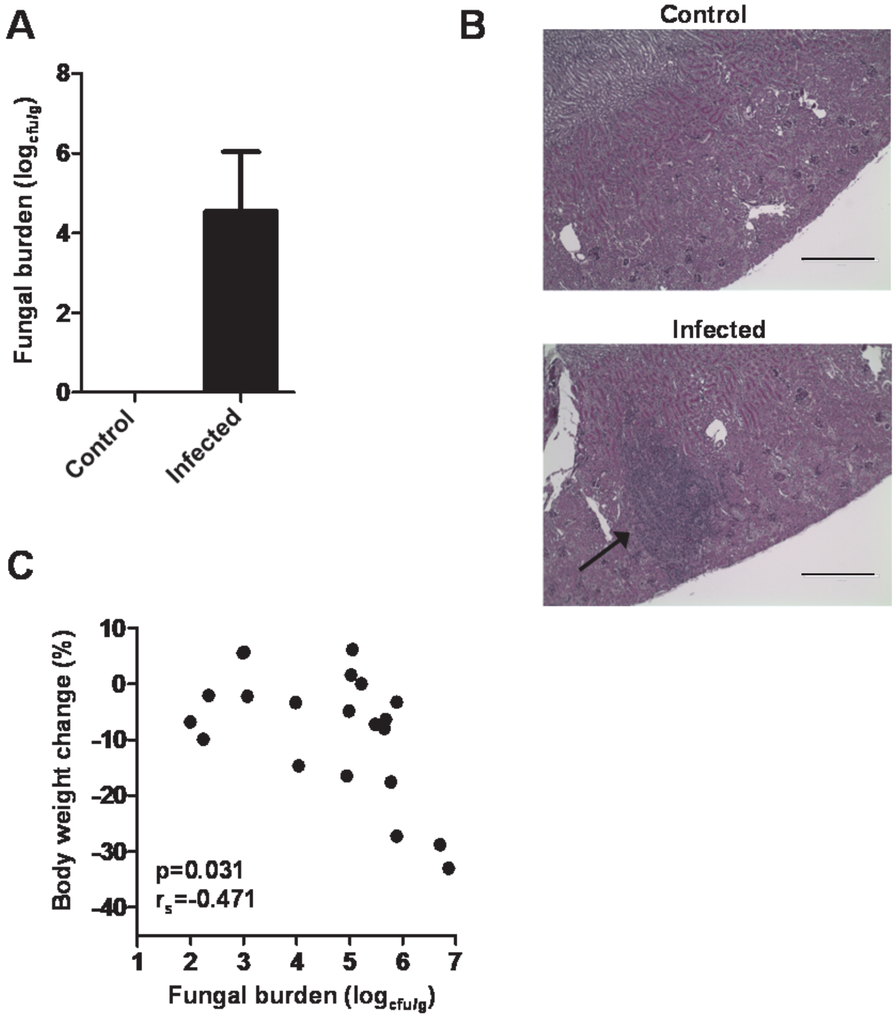

Figure 1. Disseminated *C. albicans* infection in the C57/BL6 mouse

(A) The mean kidney fungal burden in C57BL/6 mice infected for 7 days with the SC5314 strain of *C. albicans* is shown compared with control uninfected animals (data are pooled from twenty-one independent experiments). (B) Representative sections from hemotoxylin and periodic acid Schiff stained longitudinal kidney section taken from a *C. albicans* infected and uninfected mouse are shown (10x objective, scale bar 400µm). The arrow marks a large area of lymphocytic infiltrate across the kidney cortex. (C) Graph showing kidney fungal burden and weight loss after 7 days of disseminated *C. albicans* infection in C57BL/6 mice. There is an inverse correlation between kidney fungal burden and body weight (data are pooled from twenty-one independent experiments, p=0.031 R_s=-0.471 Spearman rank correlation test).

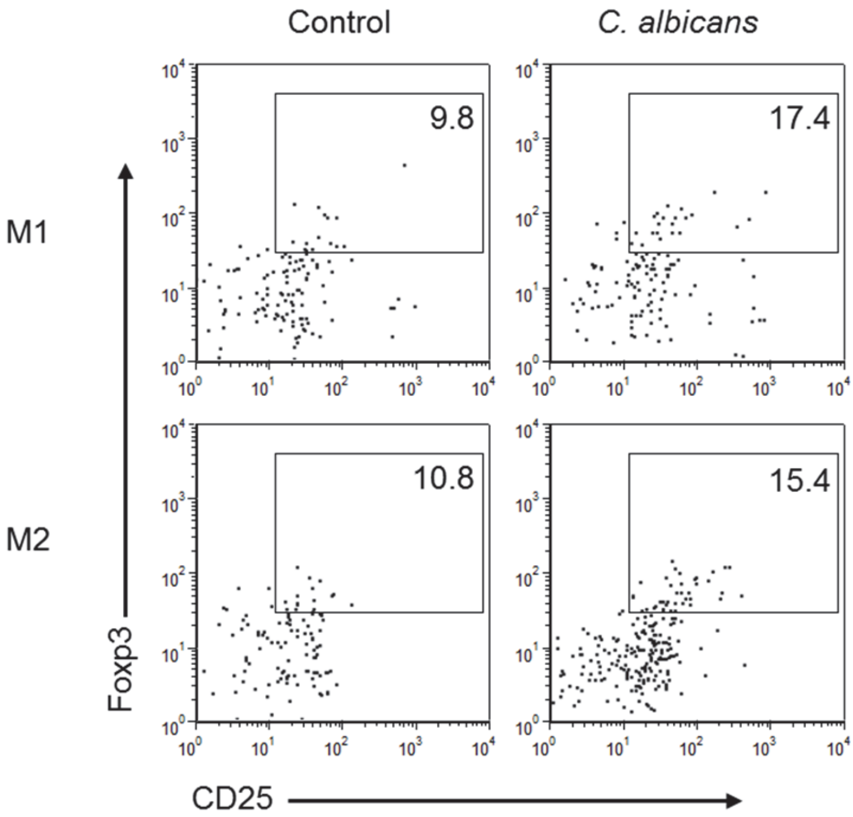

Figure 2: Kidney resident Foxp3$^+$ T cells

The percentage of CD4$^+$ cells, isolated *ex vivo* from the kidneys of control or *C. albicans* infected C57BL/6, that express both CD25 and Foxp3 is shown (cells were selected by a live and CD4$^+$ gate, M=mouse) (n=2).

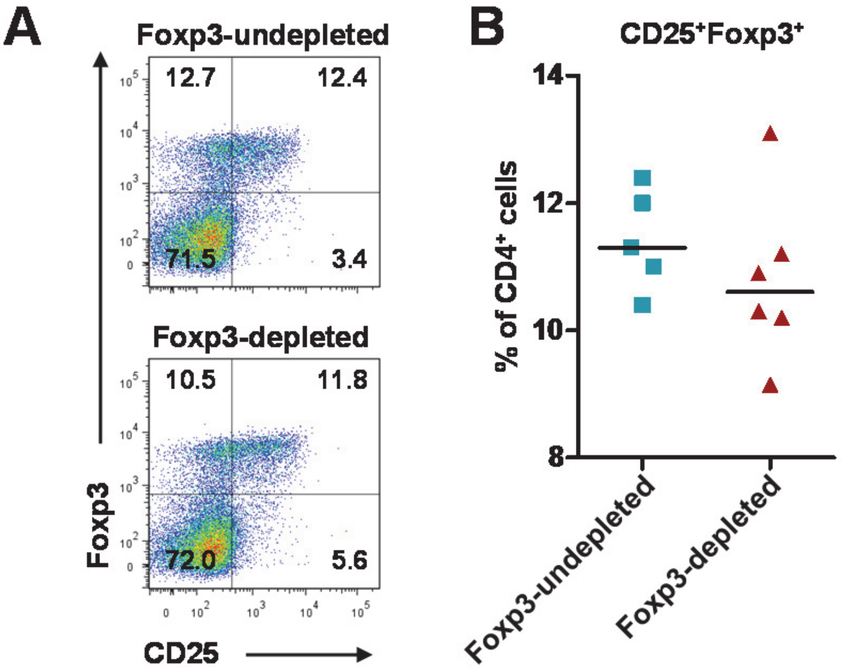

Figure 3: Depletion of Foxp3^{+} cells in Foxp3^{hCD2} mice

(A) A representative flow cytometry plot and (B) summary graph showing the populations of splenic CD4^{+}CD25^{+}Foxp3^{+} cells isolated *ex vivo* from either Foxp3-depeleted or Foxp3 undepleted mice after 7 days of disseminated *C. albicans* infection (data pooled from five independent experiments).

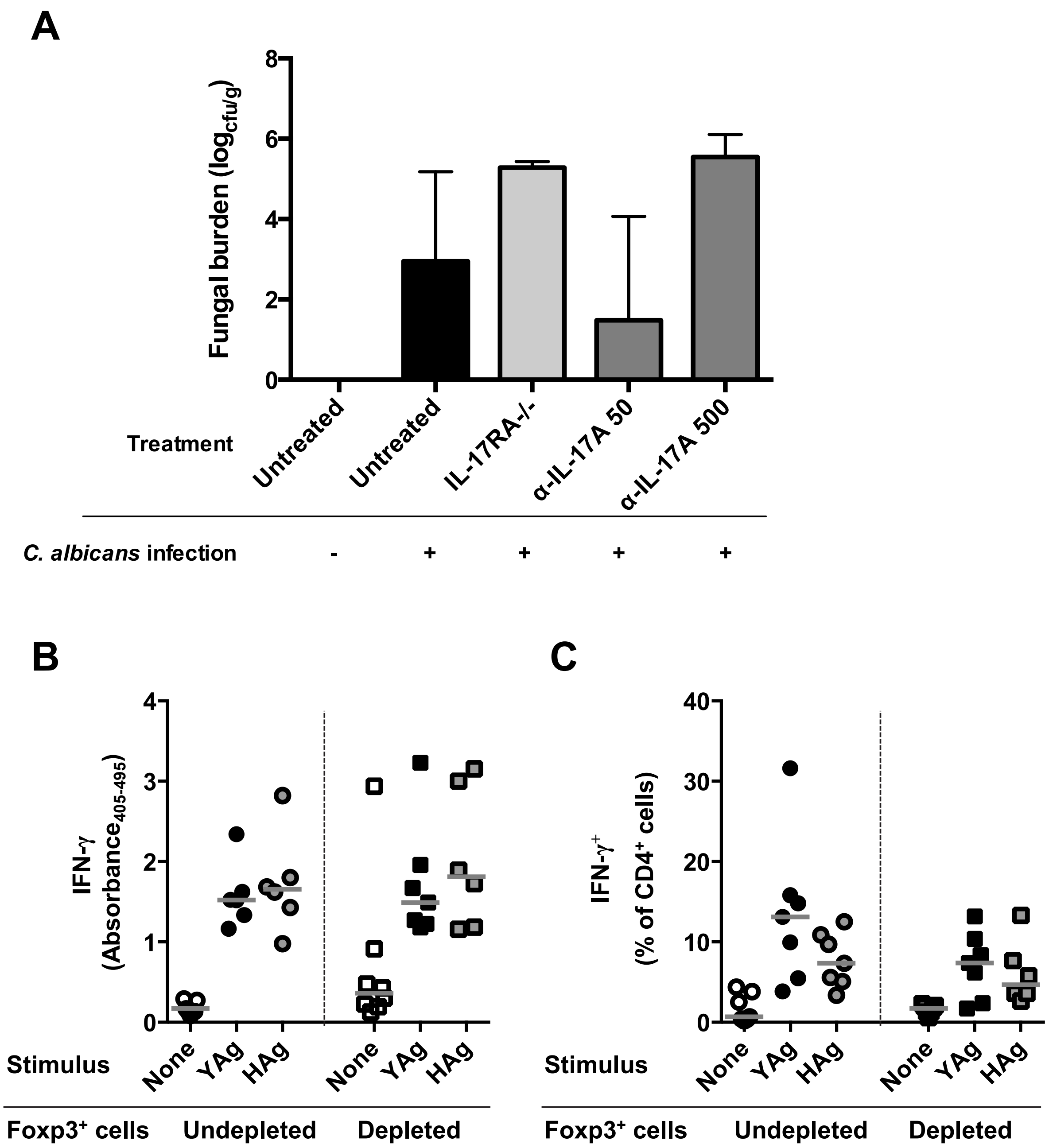

Figure 4: Depletion of Foxp3$^+$ cells alters Th17 but not Th1 responses

(A) The mean fungal burden in mice pre-treated with anti-IL-17A antibody and then infected for 7 days with *C. albicans* (data pooled from two independent experiments). Summary graphs showing (B) IFN-γ production and (C) CD4$^+$IFN-γ$^+$ populations in splenocyte cultures from Foxp3-depleted and Foxp3-undepleted stimulated with *C. albicans* YAg or HAg (data pooled from at least seven independent experiments, *p<0.05 **p<0.01 Kruskal-Wallis and posthoc Dunn's multiple comparison tests).

Materials & Methods

IL-17A depletion *in vivo*

C57BL/6 or IL-17RA$^{-/-}$ mice were given an intraperitoneal injection of 50µg or 500 µg anti-IL-17A antibody (R&D Systems, clone 50104) per mouse one day before infection with *C. albicans*, and for two days after infection (three doses in total).
