# Supplementary material for: Expansion of Foxp3+ T-cell populations by Candida albicans enhances both Th17-cell responses and fungal dissemination after intravenous challenge
Source: Eur J Immunol. 2014 Feb 13;44(4):1069–83. doi: 10.1002/eji.201343604 (PMC3992851; doi:10.1002/eji.201343604)
Supplement: Supplementary file 2 [file eji0044-1069-sd2.pdf]

European Journal of Immunology

Peer review correspondence

Expansion of Foxp3^{+} T-cell populations by *Candida albicans* enhances both Th17-cell responses and fungal dissemination after intravenous challenge

Natasha Whibley, Donna M MacCallum, Mark A Vickers, Sadia Zafreen, Herman Waldmann, Shohei Hori,6Sarah L Gaffen, Neil AR Gow, Robert N Barker, Andrew M Hall

Corresponding author: Dr. Andrew M. Hall, Division of Applied Medicine, Institute of Medical Sciences, University of Aberdeen, Foresterhill, Aberdeen, AB25 2ZD, UK

Review Timeline:	Submission date:	8 April 2013
	First Editorial decision:	3 May 2013
	First Revision received:	15 November 2013
	Accepted:	9 January 2014

Handling Executive Committee member: Prof. Caetano Reis e Sousa

Please note that the correspondence below does not include the standard editorial instructions regarding preparation and submission of revised manuscripts, only the scientific revisions requested and addressed.

First Editorial Decision – 3 May 2013

Dear Dr. Hall,

Manuscript ID eji.201343604 entitled "The expansion of Foxp3+ T cell populations by *Candida albicans* enhances Th17 responses and fungal dissemination after intravenous challenge" which you submitted to the European Journal of Immunology has been reviewed. The comments of the referees are included at the bottom of this letter.

A revised version of your manuscript that takes into account the comments of the referees will be reconsidered for publication. You should also pay close attention to the editorial comments included below.

Please note that submitting a revision of your manuscript does not guarantee eventual acceptance, and that your revision will be re-reviewed by the referees before a decision is rendered.

You can upload your revised manuscript and submit it through your Author Center by clicking on the link below.
http://mc.manuscriptcentral.com/eurjimmu?URL_MASK=jhNdXNH3Hmy5Z9DsbTCj

European Journal of Immunology

Alternatively, you can log into http://mc.manuscriptcentral.com/eurjimmu and enter your Author Center, where you will find your manuscript title listed under "Manuscripts with Decisions."

When submitting your revised manuscript, you will be asked to respond to the comments made by the referees in the space provided; however, the journal prefers that a separate point-by-point reply that includes, in full, the original comments of the referees is uploaded as a manuscript file (step 6 of the re-submission procedure) as this permits the inclusion of Figures and formatting. Please state “Reply uploaded as manuscript file” in the required section “Response to Decision Letter”. When uploading this file, you should select 'Manuscript files for review', File Designation 'Main document' and select it as the first file of the main document. This will ensure that the reply appears before the text of the paper making it easy for the referees to find. The file should be clearly labelled as a reply to the referees. In addition to providing the point -by-point reply, you should also indicate the changes made in the manuscript itself either by using the track change mode in Word or by changing the script colour of the revised sections. Please also supply a clean (non-highlighted version) that can be used for production purposes.

Please review the attached document listing important information regarding file formats and quality.

*** PLEASE NOTE ***
All pertinent correspondence relating to the peer review process of research articles, including the decision(s), the reviewers’ comments, your point-by-point response(s), and any appeal(s) that may arise, will be published online as Supporting information if the article is accepted for publication. WE THEREFORE ASK YOU TO BEAR THIS IN MIND IN THE LANGUAGE OF YOUR POINT-BY-POINT RESPONSE.

If you do not wish the peer review information to be published with your manuscript, please let the editorial office (ejied@wiley.com) know. Please note that opting out has no bearing on the peer review process or the handling of your manuscript; this has been introduced as a benefit for authors to ensure that reviewers write responsible reports that stand up to public scrutiny and to demystify the peer review process for readers. For further information, please see the Editorial entitled "Fair play at EJI" by Prof. Andreas Radbruch (http://onlinelibrary.wiley.com/doi/10.1002/eji.201370012/pdf). If you want any further information or would like to comment on this, please contact the editorial office (ejied@wiley.com).

IMPORTANT: We have your original files. When submitting (uploading) your revised manuscript, please delete the file(s) that you wish to replace and then upload the revised file(s).

European Journal of Immunology

If the revision of the paper is expected to take more than three months, please inform the editorial office. Revisions taking longer than six months may be assessed by both the original and new referees to ensure the relevance and timeliness of the data.

Once again, thank you for submitting your manuscript to European Journal of Immunology and we look forward to receiving your revision.

Yours sincerely,
Karen Chu

Dr. Karen Chu
Editorial Office
European Journal of Immunology
e-mail: ejied@wiley.com
www.eji-journal.eu
****

Reviewer: 1
Comments to the Author
This manuscript describes the effects of *C. albicans* on Foxp3+ T cells during disseminated infection. In general, the manuscript is well-written and interesting, and many of the results justify the conclusions.
Specific comments:

1. Fig. 2. In the upper left hand panel of this figure, it is indicated that exposure to YAg caused a significant increase in cell proliferation. However, the data points overlap and the median values are almost identical. The statistical analysis should be repeated.

2. Stacked bar graphs in Figs 3B and C, 5B-D, and 6F. Need to have statistical analysis, and most of the differences in Fig. 3B and C do not appear to be statistically significant.

3. Spleen vs. kidney. A significant limitation of the work is that the authors measured the immune response in the spleen (using splenocytes), yet measured the outcome of infection in the kidney. Thus, they assumed that the Foxp3+ T cells had the same phenotype in the spleen and the kidney. This assumption may not be true (for example see Spellberg, Infect Immun. 2003;71:5756-64). It would significantly strengthen the manuscript to show that Foxp3+ T cells are actually present in the kidneys of infected mice and that the presence or absence of Foxp3+ T cells alters the levels of IL-17 in the kidney.

European Journal of Immunology

4. Fig. S2. Although the manuscript contains numerous figures, I think that it is important to move Figure S2 to the body of the manuscript because it demonstrates the biological relevance of Foxp3+ T cells.

Reviewer: 2

Comments to the Author

In this study Whibley et al. showed that *C. albicans* drives expansion of Treg cells, and that there is a correlation between levels of these cells and fungal burden. They reported that *C. albicans* expands at least three populations of Foxp3+ T cells; including nTregs, iTregs and Teff. Through Tregs depletion in vivo, they showed a decrease in IL-17 production and, as consequence, increased resistance to the disease.

Overall, the manuscript is of interest because of a current debate about the role Treg and Th17 cells during infection by pathogens as *C. albicans*, but more investigations are necessary to understand the contribution of both cells for disease pathology. I felt that many results showed are only confirmatory and that the title of the MS is not adequate. It was not demonstrated that Foxp3+ T cells are in fact supporting Th17 cells or if they are the source of IL-17.

Major concerns:

1- The first part of the study (Figures 1 and 2) is showing that *C. albicans* infection induces the expansion of Tregs, suppresses Th1 response, but enhances IL-17 production. These data are only confirmatory (ref 11). The unique new data is in the Fig. 1F and G, showing that depletion of Treg leads to reduced fungal burden and inflammatory score. I would expect that less Treg could result in increased inflammation. It is hard to reconcile that the depletion of Treg results in decreased inflammatory score, decreased fungal burden, decreased IL-17 levels and increases IFN-gamma production (Fig 1 and 2). The data showing IFN-g in vivo may help to solve this question.

2- In Figure 1 the fungal burden and inflammatory score were assessed at 7 days after the infection with *C. albicans*, while in supporting information Fig 2, the effect of in vivo Treg depletion was measured on day 3 after infection. As the depletion of Tregs was done one day prior to administration of *C. albicans*, it would be necessary to show the levels of Treg in the spleens on day 7 of infection.

3- The figures 3, 4 and 5 show that *C. albicans* expands three populations of Foxp3+ T cells (nTreg, iTreg and T eff). It is not clear that Helios and neuropilin-1 are specific phenotypic markers of nTreg and iTreg. Moreover, the Fig. 3 shows that effector cells acquire Foxp3 expression following *C. albicans* infection. As

said in the discussion, effector cells can transiently express Foxp3, but it is not clear if they are able to suppress the immune response. This issue has to be clarified.

4- The Figure 3C shows that Foxp3+CD2- T cells expand ex vivo after antigen-specific stimulation. It is not possible to conclude that by the data shown (there is no statistical analysis; to me, the data are not statistically significant).

5- The figures 6 and 7 show that Treg produces IL-17 and that Treg contributes to increase of Th17 response. The role of IL-17 concerning to the control of fungal burden is not clear. Its production is very low (figure 7B and C). What was not shown is that the reduction of IL-17 production, as a consequence of Treg depletion, is responsible for the control of fungal infection. It could be due to IFN-g production. Depletion of IL-17 could be useful to clarify this subject. The mechanisms involved with Treg and Th17 cells expansion during the infection are also unclear.

6- Data previously published showed that Th17 is protective during *C. albicans* infection. As stated before, it is necessary to show the role of IL-17 during the infection.

7- The statistical analysis should be clarified. It is not showed in the figure legends. The Mann-Whitney test should not be used to compare more than two independent groups. Also, the figure legends are poorly written and insufficient to understand the experiment performed.

Minor points:

1. The abbreviation of GITR is not correct.
2. In the figure “Depletion of FoxP3 cells decrease Th17 responses in *C. albicans* infected mice”, the order of the figures mentioned is misplaced.
3. The histogram showed in Figure 7D shows CD4 T cells instead Rorgt.
4. The References section should be revised, once several words that must be in capital letters are not.

Reviewer: 3
Comments to the Author
The expansion of Foxp3+ T cell populations by *Candida albicans* enhances Th17 responses and fungal dissemination after intravenous challenge

Natasha Whibley, Donna M MacCallum, Mark A Vickers, Sadia Zafreen, Herman Waldmann, Shohei Hori, Neil AR Gow, Robert N Barker and Andrew M Hall

The authors Whibley et al., have demonstrated that antigens derived from *C. albicans* leads to the expansion of CD4+Foxp3+ Tregs respectively. They claim that CD4+Foxp3+ T cells arose from proliferation of natural Tregs and conversion of Foxp3- cells. They have further shown that the expanded Foxp3+ T cells inhibited Th1 and Th2, but promoted Th17 responses in mouse intravenous challenge model of disseminated *C. albicans* infection, and thus exacerbating disseminated candidiasis pathology in vivo. The authors deserve credit for well-controlled experiments and clearly written manuscript.

Comments:

Th17 cells have been shown to be important for clearing the fungal infection in the context of mucosal Candidiasis and depletion of Th17 cells has been known to increase the susceptibility Oral Candidiasis HIV+ individuals (6,11,23). However, an excessive inflammatory response induced by Th17 cells appears to cause immune pathology associated with disseminated *C. albicans* infection. The study here pertains to the second scenario, where Th17 cells do not play a protective role, but a negative role during infection. Both in the in vivo and in vitro models, the authors have elegantly shown that Tregs reduce Th1 and Th2 cytokines along with IL-2. Importantly, Tregs enhance the IL-17 production, which likely increases fungal burden and causes pathology. Here is an example showing that Tregs play alternative roles depending on the cytokine milieu, context of infection and location, and have not necessarily evolved to control inflammation. The results are written clearly, and experiments are performed with sufficient controls.

Minor comments:

1) The difference between the Control and Infected groups in the Figure- 5E is very moderate. Authors should perform a statistical test and show p- value for this data.
2) One unresolved question in this study is how Tregs induce IL-17 production. If the authors suggest that induction of Th17 and suppression of Th1/Th2 cytokines may involve loss of IL-2, what do they perceive to observe if they perform the infection experiments, along with exogenous IL-2 injections?

<u>First Revision – authors' response – 15 November 2013</u>

Point-by-point response

Reviewer 1

The referee is generally supportive, and makes a number of specific comments.

1. Fig. 2. In the upper left hand panel of this figure, it is indicated that exposure to YAg caused a significant increase in cell proliferation. However, the data points overlap and the median values are almost identical. The statistical analysis should be repeated.

European Journal of Immunology

We apologise that the statistical test in Figure 2 (now Figure 3) was not clear. Simple median values do not differ markedly, but the data points are paired, and so we used a posthoc Wilcoxon signed rank test. This has now been clarified both within the text and figure legend.

2. Stacked bar graphs in Figs 3B and C, 5B-D, and 6F. Need to have statistical analysis, and most of the differences in Fig. 3B and C do not appear to be statistically significant.

We agree that statistical analyses of the size of each cell population would be helpful. Therefore, we have increased the power of the study with additional experiments and demonstrated that the observed changes are statistically significant. To present the data more clearly, Figures 3B, 3C, 5B, 5C and 5D (now Figures 4B, 4C, 6B, 6C and 6D) have been changed to dot plots and the significant differences highlighted.

3. Spleen vs. kidney. A significant limitation of the work is that the authors measured the immune response in the spleen (using splenocytes), yet measured the outcome of infection in the kidney. Thus, they assumed that the Foxp3+ T cells had the same phenotype in the spleen and the kidney. This assumption may not be true (for example see Spellberg, Infect Immun. 2003;71:5756-64). It would significantly strengthen the manuscript to show that Foxp3+ T cells are actually present in the kidneys of infected mice and that the presence or absence of Foxp3+ T cells alters the levels of IL-17 in the kidney.

This paper focuses on systemic *C. albicans* infection, and we chose appropriate organs for different types of analysis. For studies of the systemic adaptive immune response, the spleen is a commonly used source of antigen experienced lymphocytes. Renal *C. albicans* burdens correlate with other symptoms of systemic infection, and since the kidney is typically the last organ to clear the fungus, provide a good indication of disease resolution (refs 9, 28). This justification has now been further clarified in the manuscript (Results Page 6 Paragraph 1). The referee raises an interesting question as to whether T cells in the spleen necessarily reflect those in inflamed organs. To address this, we have now characterized Foxp3$^+$ T cells in the kidney, and show that, as in the spleen, their numbers are seen to increase in systemic *C. albicans* infection (additional Supporting Figure S2). However, we were unable to retrieve sufficient viable T cells from the kidney for functional assays of their response to specific antigen (the paper cited by the referee does not attempt to do this, but instead reports cytokine levels of whole kidney homogenates, which is a technically simpler, but less informative, approach). These issues are now included in Discussion (Page 13, Paragraph 1) and the reference cited (Ref 36).

4. Fig. S2. Although the manuscript contains numerous figures, I think that it is important to move Figure S2 to the body of the manuscript because it demonstrates the biological relevance of Foxp3+ T cells.

We agree with this helpful suggestion, and have split Figure 1 into two, with data previously in S2 now included in a revised Figure 2, renumbering the succeeding Figures 3 to 8.

Reviewer 2

The reviewer acknowledges that our paper is of interest and contributes to the current debate about the roles of Treg and Th17 cells. However, as will be covered in more detail below, we respectfully disagree that the work is "only confirmatory", since it makes a genuinely novel contribution to the field by examining, for the first time, the roles of Foxp3$^+$ T cells in systemic *C. albicans* infection *in vitro* and *in vivo*. Other work has, by contrast, focused on mucosal infection, not tested specific responses to fungal antigen and relied on only CD25 rather than Foxp3 to manipulate T cell populations. The referee also introduces some

European Journal of Immunology

confusion about the use of such markers, since many comments appear to equate "Treg" with Foxp3$^+$ expression, whereas the diversity of Foxp3$^+$ populations is a central point of the paper.

The reviewer also felt that "the title of the MS is not adequate" because we had failed to demonstrate that Foxp3$^+$ T cells support Th17 responses or produce IL-17. In response, we believe that the paper does present clear evidence that IL-17A responses are enhanced by Foxp3$^+$ T cells both *in vitro* (Figure 3) and *in vivo* (Figure 8). The *in vivo* data are particularly powerful in this regard, and, indeed, the referee does acknowledge (in Point 5) "that figures 6 and 7 (now 7 and 8) show that Treg produces IL-17 and that Treg contributes to increase of Th17 response". We certainly agree that "more investigations are necessary to understand the contributions of both cells for disease pathology" but the current manuscript makes an important step forward that now helps to frame the questions for such future studies.

1. The first part of the study (Figures 1 and 2) is showing that C. albicans infection induces the expansion of Tregs, suppresses Th1 response, but enhances IL-17 production. These data are only confirmatory (ref 11).

   The unique new data is in the Fig. 1F and G, showing that depletion of Treg leads to reduced fungal burden and inflammatory score. I would expect that less Treg could result in increased inflammation. It is hard to reconcile that the depletion of Treg results in decreased inflammatory score, decreased fungal burden, decreased IL-17 levels and increases IFN-gamma production (Fig 1 and 2). The data showing IFN-g in vivo may help to solve this question.

To address the first part of this point, there are a number of key differences between the current study and the previously published work cited by the referee (Pandiyan et al, Reference 11). First, much of the data in Reference 11 describe the basic relationships between different T cell phenotypes outside any context of infection, and when a *C. albicans* challenge is given, it is via a mucosal route. In contrast, we focused on disseminated rather than superficial infection, in which different factors are very likely to determine pathology. Secondly, Pandiyan et al do not in fact report any expansion of Treg or Foxp3$^+$ populations by *C. albicans* antigens, either *in vitro* or *in vivo*. Finally, the experiments that involve *C. albicans* infection in Reference 11 concern the effects of adoptive transfer or depletion of CD25$^+$ cells in immunodeficient mice, whilst we selectively depleted Foxp3$^+$ cells from mice with an otherwise intact immune system. Therefore, our data, including results in renumbered Figures 1-3, do not recapitulate existing literature, but instead shed important new light on the role of T cells in fungal infection. The differences between our work and prior studies are now covered in more detail in Discussion (Page 16, paragraph 1).

In second part of this comment, we are pleased that the referee acknowledges the novelty of the data (now in Figure 2) describing the beneficial effects for the host of depleting Foxp3$^+$ cells *in vivo*. However, we wish to emphasize that we are here investigating the effects of Foxp3$^+$ cells, not exclusively Treg. One major lesson from our work, and that of our collaborators, is that Foxp3$^+$ T cell populations are heterogeneous, and include cells with effector functions that may evoke immune pathology. In this case it is entirely reasonable to posit that IL-17A production associated directly or indirectly with "plastic" Foxp3$^+$ T cells could exacerbate inflammatory damage. However, the referee is entirely correct in drawing attention to the possible role of Treg in suppressing protective IFN-γ responses as an alternative explanation that would benefit from further investigation. We had already demonstrated *in vitro* that Foxp3$^+$ populations exert opposing effects on IFN-γ and IL-17A production (renumbered Figure 3) and, prompted by the referee, we have now also tested whether the depletion of Foxp3$^+$ cells *in vivo* enhances IFN-γ responses to *C albicans* antigens. Newly included Supporting Figure S4 panels B and C demonstrate that there is no such effect on IFN-γ responses, strengthening the argument that the amelioration of fungal burden and inflammation that follows Foxp3$^+$ depletion is mediated by the reduction in IL-17A production. Discussion of these data is now included (Page 15, paragraph 2).

European Journal of Immunology

2. In Figure 1 the fungal burden and inflammatory score were assessed at 7 days after the infection with *C. albicans*, while in supporting information Fig 2, the effect of *in vivo* Treg depletion was measured on day 3 after infection. As the depletion of Tregs was done one day prior to administration of *C. albicans*, it would be necessary to show the levels of Treg in the spleens on day 7 of infection.

We are grateful to the referee for drawing attention to the relative timings of *in vivo* depletion, infection and assays. The kinetics of the loss and recovery of the $Foxp3^+$ populations when $B6.Foxp3^{hCD2}$ reporter mice are treated with depleting anti-hCD2 antibody are described by co-authors Herman Waldmann and Shohei Hori in Reference 29. We deliberately chose the most potent depleting regimen consisting of a single dose of antibody that efficiently removes the cells for up to seven days, after which their numbers recover. The alternative, repeated dosing, regimen results in a less effective depletion over a longer time period, and so we considered it less appropriate for this study of primary infection. We originally illustrated the potency of the depletion at three days, and now include later data for completeness, showing $Foxp3^+$ cells at seven days in revised Supporting Figure S3. A consideration of the merits of our depletion protocol is now included (Results, page 7, paragraph 1).

3. The figures 3, 4 and 5 show that *C. albicans* expands three populations of Foxp3+ T cells (nTreg, iTreg and Teff). It is not clear that Helios and neuropilin-1 are specific phenotypic markers of nTreg and iTreg.

   Moreover, the Fig. 3 shows that effector cells acquire Foxp3 expression following *C. albicans* infection. As said in the discussion, effector cells can transiently express Foxp3, but it is not clear if they are able to suppress the immune response. This issue has to be clarified.

The referee raises a number of good points here. First, we accept that no single marker can definitively distinguish nTreg from iTreg, or indeed Treg from Teff. For this reason we used combinations of those that have been most widely reported as being informative. Helios was the first marker that was claimed to identify nTreg from iTreg, and but since its reliability has since been questioned, other workers now advocate use of NRP-1. We found that these markers identified similar populations, and, in further support of their utility, also report that the putative iTreg cell subset with low staining includes, as predicted, most cells with evidence of “plastic” Teff characteristics. We have now highlighted the debate over the identification of nTreg and iTreg in the Discussion (page 13, paragraph 2).

The next question, as to whether effector cells that transiently express Foxp3 are suppressive, has been answered by co-authors Herman Waldmann and Shohei Hori in Reference 19. One of their major findings is that $Foxp3^+$ T cells lacking other Treg markers (such as GITR used here) have effector, and not regulatory properties. This is discussed in the paper (Page 15, Paragraph 2).

4. The Figure 3C shows that Foxp3+CD2- T cells expand ex vivo after antigen-specific stimulation. It is not possible to conclude that by the data shown (there is no statistical analysis; to me, the data are not statistically significant).

As in the response to Referee 1, we agree that statistical analyses of the size of each cell population would be helpful. Therefore, we have increased the power of the study with additional experiments and demonstrated that the observed changes are statistically significant. To present the data more clearly, Figures 3B, 3C, 5B, 5C and 5D (now Figures 4B, 4C, 6B, 6C and 6D) have been changed to dot plots and the significant differences highlighted.

European Journal of Immunology

5. The figures 6 and 7 show that Treg produces IL-17 and that Treg contributes to increase of Th17 response. The role of IL-17 concerning to the control of fungal burden is not clear. What was not shown is that the reduction of IL-17 production, as a consequence of Treg depletion, is responsible for the control of fungal infection. It could be due to IFN-g production. Depletion of IL-17 could be useful to clarify this subject. The mechanisms involved with Treg and Th17 cells expansion during the infection are also unclear.

One interpretation of these data, discussed on Page 15 and Page 16, is that, whilst IL-17A responses can provide important protection against *C. albicans*, high levels of the cytokine in the context of systemic infection can drive immune pathology and be counter-productive for the host. Experiments to test this hypothesis definitively are beyond the scope of this paper, but we have included two sets of new supporting data. First, these confirm a positive correlation between fungal burden and IL-17A production by T cells (Figure 8A). They also illustrate that, as predicted, IL-17R knockout mice, or animals receiving high doses of neutralizing anti-IL-17A antibody are more susceptible to disseminated infection, but that low levels of the antibody have, if anything, a moderate protective effect (Supporting Figure S4A). It is also plausible, as the referee suggests, that the effects of manipulating Foxp3 populations *in vivo* are mediated through their suppression of IFN-γ responses. However new data included to address Point 1 (Supporting Figure S4 panels B and C) are at odds with this view, since they show no effect of Foxp3^{+} cell depletion *in vivo* on IFN-γ responses (see also Discussion Page 15 Paragraph 2).

6. Data previously published showed that Th17 is protective during C. albicans infection. As stated before, it is necessary to show the role of IL-17 during the infection.

This question is dealt with in the response to Point 5 above.

7. The statistical analysis should be clarified. It is not showed in the figure legends. The Mann-Whitney test should not be used to compare more than two independent groups. Also, the figure legends are poorly written and insufficient to understand the experiment performed.

We acknowledge that the statistical analyses were not clear, and so these have been revised, with descriptions now included in each figure legend as well as in the text. Appropriate tests are now applied throughout, with, for example, Kruskal-Wallis and post-hoc Dunn's multiple comparison used for significant differences involving more than one independent group. Figure legends have also been revised where necessary to improve clarity.

Minor points:

1. Abbreviation of GITR corrected
2. The order of Figures has been changed
3. The labelling of the panel (Figure 8E) has been corrected
4. The Reference section has been revised

Referee 3

We are grateful to the reviewer for the many positive comments about the quality of our experiments and writing. Two minor observations are made.

1. The difference between the Control and Infected groups in the Figure- 5E is very moderate. Authors should perform a statistical test and show p- value for this data.

European Journal of Immunology

We have performed additional experiments to increase power and have shown a statistical difference in the data presented (now in renumbered Figure 6E).

2. One unresolved question in this study is how Tregs induce IL-17 production. If the authors suggest that induction of Th17 and suppression of Th1/Th2 cytokines may involve loss of IL-2, what do they perceive to observe if they perform the infection experiments, along with exogenous IL-2 injections?

The referee suggests an interesting next line of work in this area. Since IL-2 stabilises Foxp3 expression, we would anticipate that exogenous IL-2 could alter the balance between the “plastic” $Foxp3^+$ and $ROR\text{-}\gamma t^+$ cells in favour of the suppressive phenotype. However, the net effect may depend on the relative contribution of $Foxp3^+ROR\text{-}\gamma t^+$ and $Foxp3^-ROR\text{-}\gamma t^+$ cells to IL-17A mediated immune clearance and immune pathology.

Second Editorial Decision – 9 December 2013

Dear Dr. Hall,

It is a pleasure to provisionally accept your manuscript entitled "The expansion of Foxp3+ T cell populations by Candida albicans enhances Th17 responses and fungal dissemination after intravenous challenge" for publication in the European Journal of Immunology.

For final acceptance, please follow the instructions below and return the requested items as soon as possible as we cannot process your manuscript further until all items (including the reviewers' comments below) are dealt with.

Please note that EJI articles are now published online a few days after final acceptance (see Accepted Articles: http://onlinelibrary.wiley.com/journal/10.1002/(ISSN)1521-4141/accepted). The files used for the Accepted Articles are the final files and information supplied by you in Manuscript Central. You should therefore check that all the information (including author names) is correct as changes will NOT be permitted until the proofs stage.

We look forward to hearing from you and thank you for submitting your manuscript to the European Journal of Immunology.

Yours sincerely,
Laura Soto Vazquez

on behalf of
Prof. Caetano Reis e Sousa

Editorial Office
European Journal of Immunology
e-mail: ejied@wiley.com
www.eji-journal.eu
